# Tuberculosis control in postcolonial South India and Southeast Asia: Fractured sovereignties in international health, 1948-1960

**DOI:** 10.12688/wellcomeopenres.10544.2

**Published:** 2018-04-17

**Authors:** Vivek Neelakantan

**Affiliations:** 1 Independent Historian, Mumbai, India

**Keywords:** tuberculosis and international health, fractured sovereignties, BCG vaccination and controversies, South India, Indonesia, the Philippines, Burma, the 1950s

## Abstract

Between 1948 and 1960, South India (Madras State) and Southeast Asia—with an emphasis on Indonesia, the Philippines, and Burma— emerged as global centres for tuberculosis control. This article attempts to situate tuberculosis control of these two regions within the broader context of transnational health. It investigates the unique ways in which tuberculosis control in South India and Southeast Asia reflected the inner tensions between the notional magic bullet approach, which focuses on specific cures to root out the cause of the disease, and a more holistic approach that relates disease prevention to overall well-being of the population. The implementation of tuberculosis control programs across South India and Southeast Asia shed light on the nature of the postcolonial state sovereignty in public health. Across India, as in Southeast Asia, the state sovereignty appertaining to the implementation of health policy was fractured, as evident in the opposition to the *Bacillus Calmette–Guérin* (BCG) vaccination. Based on a wide range of archival materials, this article situates tuberculosis control within the context of nationalist discourse and preventive medicine. In doing so, it adds not only to the historiography of tuberculosis in non-Western contexts, which has hitherto focused on India, Sri Lanka, or Africa but also to the relatively new field of Southeast Asian medical history.

## Introduction

Tuberculosis (TB) is an infectious disease caused by *Mycobacterium tuberculosis*. It typically affects the lungs, but can affect other organs as well. The disease is spread in the air when people who are infected with pulmonary tuberculosis expel bacteria by coughing. TB is a major global health problem today. It is the ninth leading cause of death worldwide, and the leading cause of death due to a single infectious agent, ranking above HIV/AIDS ^1^. In 2016, there were an estimated 1.3 million TB deaths among HIV-negative people and 10.4 million people fell ill with the disease ^2^. The most common method for diagnosing TB remains sputum smear microscopy, in which bacteria observed in sputum samples are scrutinised under a microscope. Without treatment, death rates remain high. During the 1940s, effective drug treatments for TB, such as isoniazid and streptomycin, were developed for the first time although *M. tuberculosis* became drug resistant (defined primarily as resistance to isoniazid by 1952) ^3^.

The ancient Indians knew pulmonary tuberculosis as *Raja Yakshma* (Sanskrit for chronic respiratory ailments), and ancient Greeks described it as phthisis (the Greek word for waning). The *Charaka Samhita* (a Sanskrit treatise on Ayurveda or traditional Indian medicine) mentions that *Chandra* (The Moon God of the Hindu pantheon) suffered from consumption as a result of a curse pronounced by his father-in-law Daksha on account of excessive attachment to his wife Rohini ^4^. Ayurvedic surgeon Susruta (6 ^th^ century BCE) noted that tuberculosis was accompanied by several complications, notably chronic cough, pain in the chest and throat, fever, pain in the joints, difficulty swallowing, spitting of blood and phlegm, loss of appetite, alteration of voice, and drooping of shoulders. Ancient Indian physicians treated the disease with emetics, purgatives, and recognised the importance of a meat-based diet in restoring the bodily humours that would aid the full recovery of patients ^5^.

By the nineteenth century, consumption was seen in new ways. In popular culture, the portrayal of consumptives—selected ostensibly by their youth and beauty—endowed the disease with a biting tragedy. The Romantic poets, most notably John Keats, perceived a correlation between tuberculosis, genius, and heightened sensibility ^6^. In 1882, the thirty-nine-year-old German bacteriologist Robert Koch, proponent of the germ theory announced his discovery of *M. tuberculosis* as the causative agent of tuberculosis. The germ theory of disease framed sufferers of tuberculosis as a public health menace, who were confined to sanatoria, which separated infectious patients from the healthy.

In 1903, Albert Calmette, who was in-charge of the Pasteur Institute at Lille, along with French veterinarian and bacteriologist Camille Guérin, undertook research on the tubercle bacillus. In 1906, they discovered that immunity against tuberculosis is established by the presence of a living, but avirulent tubercle bacilli, in the body. Fifteen years later, in 1921, the BCG vaccine (named after Calmette and Guérin) was created. In Scandinavia, the extensive use of BCG vaccination as a preventative measure, accompanied by improvement in living and working conditions during the twentieth century, brought about the greatest decline in tuberculosis ^7^.

Between 1943 and 1944, during World War II, tuberculosis ravaged Yugoslavia, Poland and other parts of Eastern Europe. In early 1945, a comprehensive effort was made by United Nations Relief and Rehabilitation Agency (UNRRA) to determine the needs of people in war-ravaged Europe. The UNRRA identified TB as a public health problem and provided emergency medical supplies to affected countries. The UNRRA’s work in TB control has subsequently been continued by the World Health Organisation (WHO) since 1948. Ludwick Rajchman (who had earlier officiated as the Head of the Health Organisation of League of Nations) was very much interested that UNICEF (United Nations Children’s Fund) played a pro-active role in post-World War II international health during the late 1940s ^8^. There was a great deal of suspicion in WHO circles about Rajchman’s intentions, especially by the USA, since he had a clearly articulated vision of socialised medicine, which advocated state intervention in public health. A major bone of contention between the WHO and the UNICEF was the use of the BCG vaccination as a prophylactic measure against TB worldwide. The WHO expressed reservations regarding the large-scale use of the BCG vaccination in anti-TB programs across the world, as the vaccine had never been used for mass immunisation across the USA, and it had a low shelf life and would deteriorate easily in a tropical climate. The French government was solidly behind Rajchman in promoting the use of BCG ^9^. The large-scale use of BCG in mass vaccinations coincided with a discovery of high incidence of TB among the world’s children.

In 1948, the UNICEF signed a Joint Enterprise Agreement with the WHO and Scandinavian societies, such as Danish Red Cross, Norwegian Help for Europe, and the Swedish Red Cross ^10^. The chief objective of the Joint Enterprise of the International Tuberculosis Campaign (ITC) was to carry out BCG vaccination of children and young adults in affected countries. Mass BCG vaccination was regarded as an emergency measure to stem the occurrence of new cases of TB. The first stage of any mass vaccination against TB was the tuberculin skin test. A person who is infected with TB bacteria is expected to mount an immune response in the skin containing the bacterial proteins and would be considered a positive reactor. Only those individuals whose skin did not mount any immune response in reaction to the tuberculin skin test (non- reactors or negative reactors) would be vaccinated.

The BCG vaccine was controversial, especially in US and Great Britain ^11^. In both countries, the approach to tuberculosis was focused on educating patients to manage their own disease through the medium of the sanatorium ^12^. The prophylactic value of BCG was not even discussed in any Tuberculosis Association meetings in Britain prior to 1935, as controlled trials related to establishing the evidence of the vaccine’s safety were lacking. Joseph Aronson and Caroll Palmer conducted controlled trials in the US that seemed to favour BCG. The trials were conducted among North American Indians between 1935 and 1941 ^13^. The study group consisted of 3007 individuals between the ages of one and twenty, who were selected through random sampling based on their negative reaction to the tuberculin test. BCG vaccine was administered to 1550 individuals (experimental group) whereas 1457 served as controls ^14^. Both groups were closely monitored for six years following vaccinations. 28 deaths were assigned to TB in the control group whereas the experimental group reported only 4 deaths ^15^. The controlled trials indicated the relative effectiveness of BCG as a prophylactic measure against TB. Aronson became an enthusiastic supporter of BCG in the US scientific circles but was a lone voice ^16^. A 1947 editorial in the *American Journal of Public Health* recommended BCG for populations exhibiting high incidence of the disease such as North American Indians and in areas where clinical facilities were minimal ^17^. Even by 1950, scientists were uncertain whether it was safe to vaccinate all individuals, regardless of whether or not they had been previously infected ^18^. Whether sensitivity to tuberculin test subsequent to BCG vaccination indicated immunity to TB? There was much uncertainty with respect to whether bovine tuberculosis contributed to TB in humans. Public misunderstandings included mistaking BCG vaccination for the tuberculin test or mistaking smallpox vaccination for BCG ^19^. As Linda Bryder notes, the chief question with reference to BCG was its relative efficacy vis-à-vis other methods of TB control such as the use of wonder-drugs particularly isoniazid and streptomycin ^20^. Vaccinations against TB became widespread in Britain in the aftermath of World War II in the context of the Labour government’s efforts to introduce greater equality in the provision of health services ^21^.

Whereas the history of tuberculosis control in post-independent South India, Sri Lanka, and South Africa, and have recently received much scholarly attention, a transnational history of tuberculosis control across South and Southeast Asia, and the ways in which tuberculosis control shaped the discourse on nation-building, have thus far been largely ignored ^22^. Likewise, stigma associated with reporting tuberculosis cases and the role of private philanthropy in tuberculosis control in the postcolonial period remains neglected ^23^.

Since the publication of Randall Packard’s *White Plague, Black Labor: Tuberculosis and the Political Economy of Health and Disease in South Africa* in 1989 ^24^, various studies have been published documenting TB control in international health contexts, the most recent being Christian McMillen’s monograph *Discovering TB: A Global History*, published in 2015; and, Niels Brimnes’ *Languished Hopes: Tuberculosis, the State and International Assistance in Twentieth Century India*, published in 2016 ^25^. Whereas McMillen adopts a transnational approach to analyse resistance to BCG campaign in Madras on one hand, and shortcomings of tuberculosis treatment in Nairobi on the other, he fails to provide the political context (decolonisation) that affected the operationalisation of tuberculosis control in newly-independent nations during the 1950s.

In his book, Brimnes argues that the history of tuberculosis exposes two incarnations of the state in India ^26^. Whereas the colonial state was largely non-interventionist in matters of tuberculosis prevention and control, the postcolonial state—infused with nationalist rhetoric—was committed to national development based on science, technology, and economic planning. In the decade after independence— with the hope that technology could circumvent questions related to the correlation between poverty and ill health—India declared an outright war on disease. However, opposition to BCG vaccine revealed weaknesses in implementation of health policy at the state and district levels. There were considerable regional differences within India that affected local responses to tuberculosis control. Brimnes’ account of tuberculosis control in India is state-centric and overlooks the role of private organisations, particularly the Tuberculosis Association of India. Likewise, his characterisation of resistance to BCG vaccination— as the tension between international intervention and national integrity—alludes to but does not elaborate the political circumstances in South India of the early 1950s, which led to the emergence of the nationalist anxiety that India had adopted a public health intervention that was of a considerably lower standard than those adopted by more developed countries ^27^.

This paper contributes to the scholarship on post-World War II international health more generally. Between 1946 and 1949, South and Southeast Asian nations declared *de facto* political independence from colonialism, starting with the Philippines in 1946. Subsequently, the process of reorganisation of health services and political conflicts (such as the partition of the South Asian subcontinent and integration of princely states into the Indian Union; the four-year Indonesian Revolutionary struggle against the Dutch, 1945–49; the Huk Balahap Rebellion, which began as a peasant insurrection in Luzon, the Philippines; the commencement of the Second Indochina War in the mid-1950s; and ethnic strife in Burma) affected the collection of vital health statistics. Therefore, this study cannot infer that the BCG campaigns across South and Southeast Asia succeeded in reducing the burden of TB. Yet, disparate archival records ranging from Government Orders of Madras State, WHO and UNICEF archives, and Indonesian and Filipino articles on TB force us to re-examine how and why the BCG campaign was implemented in South India (Madras state), and Southeast Asia (Indonesia, the Philippines, and Burma) between 1948 and 1960 ^28^. This research also examines the tensions in South and Southeast Asian public health between a narrow biomedical approach that focused on the control of individual diseases and a holistic approach that linked public health with broader questions related to nation-building.

## Tuberculosis and national enfeeblement: The case of India

Every attempt to single out the social determinants contributing to the incidence of tuberculosis in colonial India must inevitably focus on the everyday experiences of millions of invisible tuberculosis patients whose lives were lived inside a constellation of social conditions that made them susceptible to the disease. These conditions included poor housing, spitting in public places, eating from a common utensil, sleeping together in the same room, shutting off openings meant for light for the sake of *purdah*, and low per-capita intake of milk and meat ^29^.

By the early twentieth century, tuberculosis in India was largely urban in its distribution. According to Arthur Lankester, a Medical Missionary from the Church Missionary Society, phthisis caused more deaths in Calcutta than either cholera or plague in 1913 ^30^. But, accurate statistics for deaths due to phthisis are not available for the early twentieth century as the disease was often misdiagnosed as fever ^31^. The incidence of tuberculosis in Bombay, Madras and Calcutta was concentrated in the mill areas of the city, where the death rates rose to over 4 per 1000 population. The migrant population of these cities consisting of students and labourers— some of whom were infected with the disease due to cramped living conditions— would return to their native villages, spreading the disease further. Young women of Calcutta between the ages of fifteen and forty— very much confined to their home due to the *purdah* system—lacked fresh air and showed a higher susceptibility to consumption than men ^32^.

Tuberculosis control in British India was largely due to the voluntary efforts of medical missionaries and local physicians. In 1939, concerned physicians and missionaries established the Tuberculosis Association of India with the help of private subscriptions and support from the King Emperor Anti-Tuberculosis Fund. It envisioned the prevention, control, and treatment of tuberculosis patients and undertook epidemiological investigations on subjects appertaining to tuberculosis. It is evident from the proceedings of the inaugural Tuberculosis Workers’ Conference convened in New Delhi (1939) that members of the Tuberculosis Association of India approached the disease from a social perspective. At this Conference, Amulya Chandra Ukil, Bacteriologist at National Medical Institute in Calcutta presented his epidemiological investigations on tuberculosis that he had carried out in a Calcutta slum. He noted that poverty influenced tuberculosis in two ways: (a) overcrowding; and, (b) undernourishment, due to families’ inability to purchase protein-rich foods, such as meat, which made undernourished people susceptible to the disease. During the 1930s, Ukil had deployed BCG vaccination to protect villagers who were seeking employment in industries and was convinced of its value as a prophylactic measure ^33^. However, the use of BCG as a prophylactic measure prior to 1948 was not widespread.

On 15 August 1945, six days after the conclusion of World War II, *The Antiseptic*—a monthly medical journal, with nationalist overtones published in Madras—contended that tuberculosis, leprosy, and cancer were the three most dangerous scourges that ravaged India. The war against tuberculosis was no less important or damaging than the war against militarist Japan ^34^. The article complimented Lady Linlithgow for encouraging the establishment of anti-tuberculosis organisations and clinics on a nation-wide basis, but warned of the effect that the provincial governments were complacent in thinking that they had solved the problem of tuberculosis by allowing private philanthropies to start clinics as and when required ^35^. The Madras public were not satisfied with half-hearted measures and were agitating for the establishment of tuberculosis sanatoria in various parts of the Presidency. Tuberculosis control formed the trope of post-World War II reconstruction in British India ^36^.

In November 1946, at the dawn of Indian independence, P.V. Benjamin, the then Technical Advisor of the Tuberculosis Association of India and subsequently TB Advisor to the Government of India, addressed the Fourth Tuberculosis Workers’ Conference in Delhi. In his lecture, he noted that the tuberculosis problem in India was a very vast one ^37^. A total of 500,000 people died every year due to this disease and 2.5 million individuals suffered from active tuberculosis, some of them lived in their homes and infected their relatives ^38^. At the time, India needed at least 4400 clinics to treat TB patients, whereas the actual number of clinics was only 120 ^39^. As India’s resources in combating tuberculosis were limited, Benjamin recommended that it was essential to train health personnel in TB work in urban conurbations.

In 1947, TB was the leading cause of mortality in Madras state. Practically one out of seven deaths in the state could be attributed to the disease ^40^. K. Vasudeva Rao, Honorary Secretary to the TB Association of Madras noted that as TB was a social disease, attributed to poverty and lack of awareness about treatment, an organised anti-tuberculosis campaign would go a long way in reducing the incidence of the disease. He noted that the high incidence of TB in Madras city and state was due to inadequacies of a rice-based South Indian diet which had a limited nutritional value, stunted the growth of the population, and produced a set of unhealthy people ^41^.

In the above paragraphs, I have contextualised the emergence of TB as a social disease in colonial India, the introduction of BCG as a prophylactic measure, and the ways in which tuberculosis became a component of post-War national reconstruction. At the time, members of the Tuberculosis Association of India were divided regarding the prophylactic value of BCG.

## The BCG conundrum in South India

This section contextualises widespread opposition to BCG vaccination in South India in the aftermath of Indian independence (1947). It also examines the fissured nature of the postcolonial state in implementing its own health policy. Whereas, as independent India’s first Governor General, at the *Sixth Tuberculosis Workers’ Conference* in Calcutta, Rajaji exhorted that physicians and Indian citizens put up a joint front against tuberculosis, four years later, as Chief Minister of Madras State, he opposed BCG vaccination ^42^. In this context, Rajaji’s paradoxical position with respect to tuberculosis control could be explained from a political perspective.

In November 1948, the Government of India and ITC signed an agreement that started a six-month BCG demonstration campaign—during which international BCG teams would train their local counterparts on effective TB prevention— a move that was primarily targeted at protecting schoolchildren from the disease. Interest of the local authorities in India in the BCG campaign varied from outright acceptance of the vaccine to resistance. The tuberculosis problem in India could be considered in its true perspective by regarding India as a continent and not as a country ^43^. There were regional differences within the country with regards to tuberculosis incidence and prevalence rates. In West Bengal, for instance, there was a good deal of misunderstanding regarding the role of the UNICEF, the ITC, and the WHO in the implementation of the anti-tuberculosis programme ^44^. The percentage of vaccinated people was miniscule. The BCG campaign in West Bengal was impeded due to opposition from private physicians who were apprehensive that the vaccination programme would interfere with their lucrative medical practice. They spread rumours among the general public that BCG vaccination was potentially dangerous ^45^. Unlike resistance to BCG vaccination, which largely dominated India’s public health landscape in 1949, the state of Travancore and Cochin presented an anomaly. With respect to tuberculin testing and vaccination, there was enthusiasm among parents and schoolchildren alike, which was lacking in other parts of India. In Travancore and Cochin, the local press sensitised the public regarding the prophylactic value of BCG ^46^.

Inaugurating the BCG vaccination campaign in Madras city on 15 February 1949, the then Governor of Madras, the Maharajah of Bhavnagar, expressed concern that even after the attainment of political independence in 1947, India had a great deal to do in raising the standard of living of the common man. One of the planks for raising standards of living was the fight against diseases, especially TB, which took a heavy toll on the population ^47^. He expressed guarded optimism that the scheme of BCG vaccination was one of the most useful weapons in the fight against TB ^48^.

The UNICEF had pledged rupees 330,000 to the Government of India for furthering preventive aspects of the campaign against TB in Madras State ^49^. Rajkumari Amrit Kaur, the then Union Minister of Health urged Madras Health Minister T.S.S. Rajan (a Congress veteran, deeply influenced by the ideals of Gandhi and Rajaji) to utilise UNICEF funding for undertaking prophylactic work against the disease in rural areas of the state ^50^. Rajan was more cautious than his colleague A.B. Shetty, who was health minister in the same government, with respect to his position on BCG vaccination ^51^. Rajan noted that medical control and supervision was essential for rolling out the mass BCG campaign and that Madras state suffered from a severe shortage of skilled medical personnel. Rajan cautioned Benjamin (TB Advisor to the Government of India) to the effect that if any untoward incident occurred in connection with the vaccination campaign, it would jeopardise Madras public opinion towards BCG ^52^. He also complained that the Madras government was trying to make both ends meet. TB control was competing with environmental sanitation and water supply for scarce financial resources. Rajan suggested that it would be more appropriate for Madras state to extend BCG vaccination, once its efficacy was empirically established ^53^.

The BCG campaign was challenged immediately by Raman. On 3 February 1949, prior to the commencement of the BCG campaign, Raman alleged that the then Health Minister of Madras State, Shetty papered over conflicting opinions of doctors, and blindly followed WHO directives to justify the launch of the BCG campaign in Madras. Shetty tried to silence critics by pointing to the success of the experimental mass vaccination against TB in Madanapalle ^54^. Shetty accused Raman of being a “hot gospeller” on the behalf of environmental hygiene ^55^. Raman’s opposition to BCG vaccination started from his idea of promoting environmental hygiene, a well-intentioned idea, which had created a scare in the minds of the people. Governments had to face opposition to the measures they introduced, whether it was Zamindari abolition, Religious Endowments Bill, or BCG vaccination ^56^. Shetty defended the vaccination campaign by stating that BCG vaccination was purely voluntary. As proof, conclusive for establishing the safety of the vaccine, he further referred to Transactions of the Commonwealth and Empire Tuberculosis Conference (London, 1947) and the International Congress on BCG (Paris, 1948) ^57^.

Raman astutely exploited disagreements among international medical experts to suggest that the vaccination against TB was still in the experimental stage. To this effect, he published the latest available results on the BCG trials ^58^. The February 1950 edition of *People’s Health* cited a French publication *La Vie Claire* on compulsory vaccination ^59^. The editor of *La Vie Claire* noted that the Soviet Five Year Plan did not seek to make BCG compulsory, but attempted to study the efficacy of the vaccine vis-à-vis other techniques of combating TB. Furthermore, the article cited one French doctor Ferru, a delegate at the First BCG Congress (1948), who inferred that BCG was only an inferior vaccine incapable of guaranteeing real protection against TB ^60^. Furthermore, Raman launched an attack in *People’s Health* on Amrit Kaur, for introducing mass vaccination in the country flippantly and irresponsibly, and against India’s opposition parties for not deriving political capital out of the popular opposition to BCG ^61^. In the March edition of *People’s Health* Raman noted, “The more the facts come to light, the more we are convinced that the ulterior motive for the campaign in this country is to use the people of this country as human guineapigs ^62^.”

Raman looked to M.K. Gandhi and the WHO Preamble—which defines health positively as complete physical, mental, and moral well-being and not merely as the absence of disease or infirmity—for inspiration while opposing the BCG campaign. In the January 1950 editorial of *People’s Health*, commemorating Republic Day, he despaired about the health conditions of newly-independent India:

The illiterate feels; he cannot think. All he knows is a change from the Union Jack to the Tri-colour, a change from Wavell to Rajaji, the substitution of *Rajpramukhs* [governors] for Highnesses, have meant no change in his health, no improvement in his living conditions—not to mention a positive deterioration in the last two years. He is in no fettle to be ecstatic about the 26 ^th^ of January this year ^63^.

By improvement of living conditions, Raman meant that the Ministry of Health fulfil minimum requirements for Indians, such as clean food, clean water, and a clean home. Raman’s relentless crusade against BCG led the Madras government to postpone mass vaccinations. *People’s Health* ceased publication in 1951. A year later, Rajaji became the Chief Minister of Madras State, a position that he relinquished in 1954.

During his tenure as Chief Minister, Rajaji disagreed with Nehruvian centralised planning and subsequently founded the Swatantra Party by 1959. From a political perspective, Rajaji’s opposition to BCG vaccination must be seen in terms of his opposition to the centralising tendencies of Delhi ^64^. At the time, in order to compensate for the shortfall in public health spending in Madras state, Rajaji mobilised private philanthropy and established the *Maharaja of Bhavnagar Kshayrog* Fund (a corpus fund instituted in the name of the former Governor of Madras, to treat indigent tuberculous patients). However, the Ministry of Health at Delhi expressed reservations with respect to the Madras Health Ministry administering a special corpus fund for treating tuberculous individuals rather than strengthening existing public health institutions. In 1954, Rajaji resigned as Chief Minister of Madras, due to his controversial educational reforms. As a fiscal conservative, he sought to reconcile between reducing the cost of primary education on one hand, and increasing the enrolment of students, on the other. He advocated to the effect that children learn the vocations of their parents after school. This policy received much censure from the opposition DMK ( *Dravida Munnetra Kazhagam*) party, which accused Rajaji of perpetuating the Hindu caste-based traditional occupation (also known as *kula kalvi thitttam* in Tamil). At the time of his resignation as Chief Minister of Madras, Rajaji developed anti-statist views, partly as a result of his disagreements with Nehruvian Big Science, and increased state intervention into the private lives of citizens ^65^.

Soon after abdicating as Chief Minister, Rajaji tapped into popular resistance to the BCG campaign that had been brewing in Madras state, since A.V. Raman’s articles critiquing the campaign appeared in *People’s Health* between 1949 and 1950. In Tanjore and Madurai districts, a large number of people would not come out for tuberculin test and BCG vaccination ^66^. In his pamphlet, “BCG Vaccination: Why I Oppose It,” Rajaji anchored his critique of the BCG vaccine in modern science, claiming:

I am not against modern “western” therapy or modern science. BCG has nothing to do with the principles of modern western therapy. If at all, it is akin to the principles of Homoeopathy. It proceeds on a creed very similar to that of homoeopathy, namely, that diseases are to be dealt with by the administration in mild forms of things that produce identical symptoms. The difference is that the homoeopath does not introduce what multiplies in the human body, but the BCG man introduces a large body of living multiplying organisms, which are intended to remain alive in the body of the patient to produce the intended result ^67^.

Rajaji marshalled arguments from *The Lancet*, and various articles from the *Journal of the American Medical Association* to substantiate his argument against BCG vaccination.

Furthermore, while opposing BCG on scientific grounds, Rajaji also noted that treatment with wonder drugs like isoniazid, although in the experimental stage produced promising results, was limited ^68^. In his booklet, Rajaji included unverified complaints of children developing infections after the intake of the BCG vaccine ^69^. All the complaints were received after he initiated opposition to BCG. The range of complaints that Rajaji received ranged from blindness, skin diseases, and mental ailments. Some of the Tamil newspapers of the time, particularly *Dina Thanthi,* reflected the general uneasiness in rural areas with mass vaccinations. In the district of Cuddalore, on 14 September 1955, schoolchildren launched a strike against the BCG, as it was alleged in the local newspaper that students were coerced into accepting the vaccine by the principal ^70^. Rajaji’s pamphlet contained ten cases highlighting tales of children taking ill and dying, but only one case highlighted that the complications supposedly induced by BCG were due to epidemic encephalitis ^71^. In order to assuage public fears regarding BCG, the Madras government issued a booklet entitled “Truth about BCG: Why Government has launched a Mass Campaign.” The booklet inferred from available statistics that whereas the tuberculin-tested population in the state was 15 million, only 22 people reported side-effects, due to BCG, indicating that the vaccine was harmless ^72^. The vaccine used in Madras state was identical to the one used in Ceylon, Malaya, and Burma, but no single case of complaint had been received from any of these countries ^73^.

The anti-BCG campaign in Madras state, led by Raman (between 1949 and 1950), and subsequently by Rajaji during the mid-1950s highlighted national anxieties that Indians were being used as experimental subjects by the WHO and international aid agencies. Both Raman and Rajaji adroitly exploited cleavages within the international community, regarding the efficacy of the vaccine.

## Fractured postcolonial health sovereignties: TB control in postcolonial Southeast Asian contexts

Tuberculosis control in newly-independent nations of South and Southeast Asia were part of the nationalist rhetoric, which associated tuberculosis as an endemic disease, and vitiated the overall productive capacity of the population. During the 1950s, the WHO assisted the governments of Indonesia, the Philippines and Burma to control tuberculosis through a horizontally structured program that sought to integrate the control of the disease into national health services. This section critically examines the organisational shortcomings of TB control in each of these countries that relied on a combination of prophylactic measures such as vaccination of infants and susceptible population, on one hand and administration of wonder drugs such as isoniazid to treat patients, on the other.

Created in 1948 as a specialised UN agency concerned with international health, the WHO had to come to terms with the fraying of the British, Dutch, and French colonial empires in Southeast Asia and escalating Cold War tensions between the Soviet Union and the US ^74^. The constitution of the WHO provides for decentralisation of operational functions to the regional offices. Each Regional Office of the WHO assists in framing national health programs in the light of local needs. The WHO definition of a regional office was supposed to aggregate nations sharing similar epidemiological profiles ^75^.

In his address to the 79 ^th^ Meeting of the American Public Health Association (1951), Chandra Mani (the first Regional Director of the SEARO from India between 1948 and 1967) noted that in Southeast Asia chronic poverty and disease had arrested national progress ^76^. TB was widespread in India and Java ^77^. He warned international agencies to the effect that large-scale campaigns against disease in Southeast Asia would be unsustainable, given the lack of funds and trained health personnel. During the 1950s, the SEARO concentrated on helping its member states in campaigns against malaria, TB, yaws, and betterment of maternal and child health, with an added emphasis on strengthening local health services. SEARO assisted countries of the region in the campaign against disease by training national health staff in new approaches to disease control through pilot demonstration projects ^78^. Likewise, between 1951 and 1955, the WPRO assisted the governments of Malaya, Brunei, Cambodia, the Philippines, Sarawak, Singapore, and Vietnam in TB control through BCG campaigns ^79^. Towards the close of the Sixth World Health Assembly (1953), delegates belonging to the Study Group on Tuberculosis were unanimous that BCG was a prophylactic tool in countries having a high burden of TB ^80^. But, a close study of articles published in *Berita Tuberculose Indonesiensis* ( *Indonesian Tuberculosis Journal*) reveal scepticism within the country’s medical establishment with respect to the efficacy of BCG in nationwide campaigns against the disease.

Subsequent to the transfer of political sovereignty to the Indonesian republic, the postcolonial state inherited a health system devastated by War in the Pacific (1942–45), and four years of revolutionary struggle against the Dutch (1945–49). The country was neither able to respond to disease outbreaks nor meet the demands of curative healthcare ^81^. Indonesia suffered from a severe shortage of physicians, estimated at 1200 for a population estimated at 70 million (1949), mostly concentrated in urban areas ^82^. The emergency program of the government covered the control of epidemic diseases (smallpox, plague, dysentery, and plague), and the prevention and treatment of endemic diseases (malaria, TB, venereal diseases, and leprosy). The anti-TB work was under the jurisdiction of the Tuberculosis Section of the Ministry of Health but was subsequently decentralised to provincial and local administrations. Johannes Leimena, the then Minister of Health (1947–1952 and subsequently from 1955–56) conceptualised TB clinics— that dealt with diagnosis of the disease, isolation, and nursing of the patient—as lynchpins in the campaign against the disease. But, due to financial difficulties in putting the scheme into practice, the Indonesian Ministry of Health prioritised BCG vaccination.

In 1951, L.G.J. Samallo, Director, TB Division of the Ministry of Health associated the emergence of TB with immiseration of the Indonesian masses under three-and-a-half centuries of Dutch colonialism. Consequently, in Samallo’s narrative, Indonesia emerged as a hungry nation ^83^. A mobilisation mentality was evident in Samallo’s assessment of the TB problem in Indonesia. He appealed to the Javanese tradition of *gotong rojong* (mutual sharing of burdens) to enlist popular support against the disease ^84^.

Between 1952 and 1960, tuberculosis control in postcolonial Indonesia was conceptualised as a social-hygienic and socio-medical problem. Conceptualising TB as a social-hygienic problem involved educating patients and their families about the infectiousness of the disease, training patients to scientifically dispose sputum, ration administration of prescribed medicines, especially streptomycin and isoniazid, and administer BCG vaccination to negative reactors. In contrast, as a socio-medical problem, Indonesian physicians perceived TB as a curable disease at the individual level—with the availability of wonder drugs— particularly isoniazid.

In 1952, the Indonesian Ministry of Health, in collaboration with the WHO Regional Office for Southeast Asia operated a pilot TB demonstration project in Bandung to investigate the prevalence of tuberculosis and suggest preventive measures.

In 1952, when the project was initially implemented, Bandung was facing an acute shortage of hospital beds. Consequently, the TB demonstration centre was forced to adopt ambulatory chemoprophylaxis—visiting patients’ homes, educating patients and their families about the infectiousness of the disease, administering drugs, such as isoniazid and streptomycin, and providing follow-up on treatment of TB patients ^85^. The Bandung TB project was administered directly by the Indonesian Ministry of Health between 1952 and 1954. However by 1954, the Ministry of Health devolved administration of the project to the Inspectorate of Health of West Java. The municipal services of Bandung city were not involved in the execution of the project as they lacked sufficient funding. The project thus suffered from administrative and financial bottlenecks. Leimena attempted to integrate TB within the framework of preventive health services in the so-called Bandung Plan (1951). However, the plan had to be aborted, due to a lack of finances, which consequently inhibited treatment and follow-up of individual TB patients.

Concomitant with the establishment of the TB demonstration centre in Bandung in 1952, the Ministry of Health also initiated mass BCG vaccination of infants. However, within a few months, the BCG campaign in Indonesia ran into technical trouble, as the vaccine caused fevers among newborn infants, under the age of one, causing Leimena to reassess the long-term benefit of using mass vaccinations against TB ^86^. At Bandung, the BCG team encountered passive resistance from the Chinese community in Indonesia, who opposed administration of the vaccine ^87^. In Jakarta and Bandung, complaints emerged that vaccine lots —imported from Manila, of varying potencies—administered to children induced fevers ^88^. In Bandung, a rumour circulated that BCG made the incipient weak ^89^. Subsequently, the WHO distanced itself from the BCG campaign by issuing a statement to the effect that it had advised Ministries of Health to exempt children under the age of one from vaccination ^90^.

The BCG campaign extended to the entire archipelago by 1956 ^91^. The campaign’s performance across Indonesia was variable. The province of North Sumatra, in particular, had among the best organised BCG campaigns in Indonesia as nearly 95% of the population was covered by tuberculin surveys ^92^. But, in Makassar, the capital of South Sulawesi province, the health authorities received complaints—of side-effects such as abscesses—from people who had undertaken the tuberculin test. Incidentally, the complaints coincided with a smallpox outbreak in Makassar in 1956 ^93^. Due to the fear associated with tuberculin testing and vaccination, and the stigma associated with the detection and isolation of smallpox cases, locals actively resisted the BCG campaign ^94^.

From the archival sources, it is unclear whether the resistance to BCG in South India influenced parallel developments in Indonesia. But, given the episodes of resistance to the vaccine under disparate circumstances, as in the Indian context, one could adduce that disagreements between WHO consultants, Indonesian physicians, and the diverse ethnic groups inhabiting the archipelago could be due to terminological and conceptual ambiguities regarding the framing of tuberculosis ^95^.

During the 1950s, as in the case of Indonesia, tuberculosis was the second leading cause of mortality in the Philippines, next only to malaria. In his opening address at the First Southeast Asia Medical Conference convened in Manila in 1951, Eugenio Alonso of the Philippine Medical Association declared that no want was more individualised than disease ^96^. The losses of income of a wage earner due to malaria or the death of a mother due to tuberculosis were individual problems that required targeted interventions ^97^. At the time, there was no consensus among Philippine physicians regarding the efficacy of mass BCG vaccination as a prophylactic measure against TB.

Between 1946 and 1951—in the aftermath of War in the Pacific— the US Public Health Services (USPHS) allotted US$ 5 million for public health reconstruction in the Philippines, of which $ 1 million was set aside for TB control ^98^. At the time, Leroy Young—a short-term USPHS consultant to the Philippine government—introduced mass chest radiography surveys that sought to determine the proportion of infected population ^99^. C. Penaflor of the Philippine Department of Health noted that the surveys were of little consequence ^100^. He was critical with respect to the inaction on the part of the Department of Health in the follow-up of the treatment of infected patients who continued to spread infections.

Even prior to War in the Pacific, BCG was used in the Philippines on a limited scale. But, Penaflor was not particularly impressed with the results of using BCG as a prophylactic and was inclined to oppose the mass program sponsored by the WHO and the UNICEF (1951) ^101^. Penaflor expressed six reservations with respect to the use of BCG in mass vaccinations ^102^. First, there was much uncertainty with respect to immunological processes triggered in the human body on account of BCG. Second, there was limited knowledge with respect to how BCG responded to standard tests in immunology. Third, why was BCG ineffective in the case of bovine TB? Fourth, whether organisms used in the vaccine were attenuated bovine bacilli? Or, were they only saprophytes such as Moeller’s Timothy Bacillus? ^103^ The properties of the BCG vaccine had not been studied since Calmette and Guerin conducted their experiments. Fifth, there was no evidence with respect to the effects of administering BCG vaccine to a person who demonstrated naturally established tuberculin allergy. Sixth, whether BCG was the only method to reduce the burden of TB? The proponents of introducing mass vaccination in the Philippines, including the Department of Health used the evidence of the WHO’s vaccination of 50 million children as evidence for the BCG vaccine’s widespread use ^104^. Penaflor contended that the WHO’s BCG campaign was implemented under disparate conditions and there was no uniformity with respect to the training of vaccinators, or the lack of uniform standards for interpreting results of the tuberculin tests. He attributed TB to poverty, malnutrition, and overcrowding. As a long-term solution to the tuberculosis problem, he prescribed a nationwide program of health education and the investment in hospital care that would facilitate the detection, isolation, and the treatment of tuberculous patients. To eliminate malnutrition, he recommended a comprehensive social security program.

Between 1948 and 1949, the Philippines became the first country in Southeast Asia to introduce freeze-dried BCG vaccine on an experimental basis ^105^. The experiment was spearheaded by Filipino physicians from the National Chest Centre, particularly Sofia Bona-De Santos. 14,898 individuals— non-reactors to the tuberculin test— were immunised with freeze-dried BCG. A follow-up tuberculin test on the vaccinated individuals after an interval of six months indicated that a little over 83% of individuals registered a vaccinial allergy, indicating immunity from TB ^106^. Based on the success of the freeze-dried BCG vaccine trial, and given the high incidence of TB among children, Filipino physicians particularly Bona-De Santos prescribed the use of BCG as a supplementary measure against the disease. Since the resources for radiography, isolation and treatment of patients, and health education were lacking, the Philippine government turned to BCG vaccination as a quick-fix solution to the TB problem. In its efforts, the government was aided by the WHO. The government steamrolled Penaflor and other critics of the BCG campaign by 1951 although scepticism towards BCG resurfaced in 1955 ^107^.

In 1951, the US Public USPHS assisted the Filipino Department of Health in establishing a laboratory at Alabang for the domestic production of BCG. The Philippines officially inaugurated its mass BCG campaign in October 1951, with financial support from the UNICEF. BCG vaccination was initiated on an experimental basis in the province of Pangasinan, in the Visayan group of islands constituting the Philippine archipelago. Public address units, supplied by the UNICEF were used by the provincial teams in Pangasinan to educate the local population regarding TB prevention. However, tuberculin testing of the population, and the administration of BCG to negative reactors, were suspended indefinitely in 1951 due to typhoons ^108^. The vaccination coverage was incomplete, as less than 50% of children under the age of six were reached ^109^.

TB control programs in the country between 1951 and 1955 primarily relied on a combination of administering BCG vaccination to tuberculin non-reactors and detecting the disease through radiography ^110^. In 1955, the Philippines initiated its first pilot TB control project in the northern province of Ilocos Norte with technical assistance from the USA, on the condition that TB control would be integrated into the rural health services ^111^. The provincial government at Ilocos Norte provided impetus to the project. Another motivating factor was President Ramon Magsaysay’s emphasis on linking TB control with extension of healthcare facilities to rural areas of the country ^112^. Physicians working in rural areas were trained to diagnose and treat TB patients. The project relied on a three-pronged approach of administering tuberculin tests and vaccinating negative reactors, detecting the disease through radiography, and treating infected individuals with isoniazid or streptomycin. BCG vaccinators collaborated with mobile chest clinics to detect TB patients. The then provincial health officer, Severo Senen enlisted the assistance of the national health staff to train rural health units in anti-tuberculosis work ^113^. The population of Ilocos Norte enthusiastically reported at the mobile chest clinics for TB detection. The provincial government subsidised drug supply to the mobile chest clinics.

By 1957, the vaccination aspect of the TB control program in Ilocos Norte underwent a change from mass vaccination to selective vaccination of vulnerable groups, particularly schoolchildren in the 7–14 age group ^114^. Between 1955 and 1958, due to effective surveillance and treatment, mortality rates attributed to TB in the province registered a precipitous decline from 178 per 100,000 deaths in 1955 to 82 in 1958 ^115^. But, gains in Ilocos Norte registered from effective case detection were neutralised by inadequacies of drug supplies ^116^. Rising costs of anti-TB medications had an adverse effect on the control program as patients were discontinuing treatment. Another setback for the Ilocos Norte project was the efficacy of the BCG vaccine itself. Many children who were BCG vaccinated were still tuberculin negative ^117^. The BCG vaccinations administered in the Ilocos Norte project were of varying potencies ^118^.

Between 1955 and 1960, Filipino paediatricians particularly M.B. Abad—after observing clinical manifestations of tuberculosis in vaccinated children— raised concerns with respect to safety of BCG ^119^. The first case related to a female infant— aged five months and vaccinated at birth—was admitted to hospital in December 1955 with stiffness of neck. After four months of hospitalisation, she was discharged with poor vision. Subsequently, at the age of two years, she was paralysed and readmitted. She was discharged against doctors’ advice and died the same day ^120^. The second case related to a male infant, aged nine months—vaccinated at birth—who was admitted with diarrhoea in May 1957 ^121^. At four months of age, he developed an axillary gland abscess. X-ray examination revealed calcification in the right lobe of the liver. The infant was treated with anti-TB drugs and was discharged. In the third case, a female infant aged—BCG vaccinated at birth and aged seven months—was hospitalised in August 1960 with fever, vomiting, and a bulging fontanelle ^122^. She had no prior exposure to TB. The tuberculin sensitivity test recorded a negative reaction. The infant was discharged after treatment. Filipino paediatricians warned to the effect that if BCG were to have prophylactic value, it had to be administered under rigid precautions such as isolating children before and after vaccination from sources of infection.

While it was one thing to use mobile X-ray units and wonder drugs like isoniazid to treat tuberculous patients, it was yet another to follow-up on treatment. Surveillance and follow-up of treated cases was a weak branch in tuberculosis control in the Philippines throughout the 1950s ^123^. The Philippine health authorities did not have accurate prevalence studies of TB throughout the archipelago when the BCG vaccination campaign was initiated in 1951. Diagnosis for TB was based only on radiological findings and not bacteriological examinations. Patients could not follow-up on treatment, as isoniazid and streptomycin were unaffordable. In the mid-1950s, the Department of Health divested disease control in the Philippines to the rural health units, who were already over-burdened with public health education, environmental sanitation, maternal and child health, and smallpox vaccination ^124^. The rural health units could not follow-up on individual cases. Between 1954 and 1958, the national mortality rate attributed to TB only registered a marginal decline from 114 per 100,000 to 104 per 100,000 individuals ^125^.

In 1948, subsequent to Burma’s independence from Britain, the country was enmeshed in a civil war between the union government and different ethnic groups, the Karen in particular and the communists. Political unrest and consequent internal displacement of population and overcrowding in urban areas like Rangoon created poverty and predisposed the migrant population towards TB. The prevalence of TB in Burma was characteristic of other SEARO member states—with a higher pool of infectivity in urban areas ^126^.

In the aftermath of Burma’s independence, as government functions expanded, there was an ad-hoc creation of ministries ^127^. The union government attempted to combine health and education under the overarching umbrella of social welfare. But, the Ministry of Social Services was rather unwieldy and by 1952, education became a separate ministry. In 1953, a new Burmese Ministry of Health and Local Government was carved out from the earlier Ministry of Social Welfare. Then, local government was split from health. Consequently, the administration of hospitals and mass vaccinations—earlier under the jurisdiction of local bodies—were placed under state control. Consequently, municipal services such as street cleaning and refuse collection in Rangoon and other big cities collapsed.

In 1952, Prime Minister U Nu introduced Burma’s first welfare plan entitled *Pyidawtha* or “Happy Land ^128^.” The main motive of the government was to enlist the support of the Burmese citizens in its ongoing campaign against the communists. At the Pyidawtha Conference (August 1952) Nu pledged to bring to every Burmese citizen a brick house, a car, and 80 kyats in salary ^129^. He also pledged to work hard to make the people healthy as the legendary Burmese heroes Kyan-Siq-Thà, and Ananda Thuriya ^130^. The task of rehabilitation of Burma’s health services during the early 1950s was complicated considering that over half of the hospitals were devastated by the civil war. Given the country’s shortage of doctors (estimated at 400 doctors for 1951), the government trained health assistants to work in rural areas ^131^. The Government identified malaria, venereal diseases, and TB as the main diseases afflicting the nation and enlisted international assistance to combat these diseases. Between 1950 and 1954, there were an estimated 300,000 TB patients in Burma ^132^.

TB control in Burma during the 1950s consisted of a two-pronged strategy, i.e. administration of BCG to non-reactors to the tuberculin test, and institutional treatment of confirmed patients. In 1951, in conjunction with UNICEF and WHO assistance, the Burmese Ministry of Health introduced mass tuberculin testing and selective administration of the BCG vaccine to non-reactors. The institutional treatment of TB patients using wonder-drugs especially streptomycin and isoniazid within the confines of the hospital or home (domiciliary treatment) with a view to reduce infection was first implemented in Rangoon (1951) and Mandalay in 1954.

In 1951, the SEARO in collaboration with the Burmese government set up a TB Training and Demonstration Centre at Rangoon. The Rangoon Centre undertook tuberculin surveys among children in various cities of Burma to determine the incidence of TB. The surveys revealed much higher rates in urban areas to which refugees had migrated. Rangoon thus revealed 76% positive reactors in the 0–15 age group in contrast to 40% in the same age group in Mandalay ^133^. The infection rate in Rangoon was much higher in children attending schools in crowded areas. B. Papanicolaou, former WHO Senior Advisor to the Rangoon Centre, noted that between 1951 and 1952, 9265 new cases of TB were detected through fluoroscopy and mass radiography ^134^. Papanicolaou’s study of TB in Rangoon indicated that the incidence of TB was high in malnourished individuals. Of 552 observed TB cases, only 68 had satisfactory housing and sanitation ^135^. In 1953, a series of disastrous fires burnt out considerable areas of Rangoon. A number of meetings were held to determine measures to prevent the spread of TB. The Rangoon Centre proposed segregation of tuberculous patients and their families in barracks. One room in the barracks would be reserved for night isolation and treatment of TB patients ^136^. But, the proposal to segregate of the families of tuberculous individuals and their families failed as there was popular opposition to the idea. Papanicolaou commented that by the early 1950s Rangoon’s population had become “tuberculosis minded,” alluding to the fact that TB patients welcomed streptomycin injections due to its ability to give symptomatic relief but disliked collapse therapy, a surgical procedure to treat TB ^137^. The Burmese government’s attempt to enlist private practitioners from Rangoon in the TB control program was met with a poor response as they were reluctant to abandon their lucrative practice.

By mid-1952, the Burmese authorities began to take increased interest in the BCG campaign. Between August 1952 and June 1953, approximately 819,230 persons were tuberculin tested and 96, 794 were vaccinated ^138^. Subsequently, the Burmese government incorporated BCG vaccinations into the routine activities of the rural and urban health centres ^139^.

In mid-1954, the SEARO assisted the Burmese government in setting up a second pilot TB control and demonstration project in Mandalay. The project emphasised TB prevention, train local personnel in modern methods in diagnosis and control of the disease, including domiciliary chemotherapy, and epidemiological surveillance of the disease. The project encountered difficulties appertaining to the recruitment of qualified Burmese medical personnel. Although the Mandalay project was successful in tracing TB patients and their contacts, scarcity of drugs particularly isoniazid hampered treatment of individual patients ^140^.

TB control in Burma was constrained by administrative and organisational setbacks. For instance, during the mid-1950s, the SEARO proposed conducting a nation-wide prevalence survey but government permission was not forthcoming. Projects sanctioned by the WHO that required funding from the Burmese government faced procedural delays ^141^. The bureaucratic hassles faced by the WHO were a part of an assertion of the Burmese government in asserting its sovereignty in matters of health.

Tuberculosis treatment and prevention across Southeast Asia during the 1950s had to compete with other public health programs, such as malaria eradication, maternal and child health, and environmental sanitation. Across South and Southeast Asia during the mid-1950s, when the BCG campaigns were well-underway, there was a noticeable gulf between citizens’ aspirations for freedom from disease and chronic poverty that inhibited disease control programs. In his short story “My Kampung,” published in 1952, when disease eradication programs in Indonesia were underway, Toer’s tones alternate between pessimism and sarcasm. He boldly declares that a “small guerrilla squad” (referring to the Indonesian revolutionaries (1945–49) are cautious and not likely to lose more than ten people in two years, but in my *Kampung* (neighbourhood), people die a “cheap death ^142^.” He then enumerates the deaths due to preventable diseases. There is one person who dies of chronic venereal disease; the mother who kills her child with an overdose of worm medicine; and the print-setter who dies of lead poisoning, and countless victims of tuberculosis. Toer implicitly mocks the utopia of a world free of disease. Toer’s short story illustrates the limitations of using disease control as a technological fix to public health problems in developing countries.

## Conclusions

Having analysed the attempts to control tuberculosis across four disparate postcolonial contexts—Madras state in South India, Indonesia, the Philippines, and Burma—during the 1950s, it is inevitable to infer that the campaigns were inconclusive. But, why? The first answer that comes to mind is the lack of reliable epidemiological data regarding the incidence and prevalence of TB, in each of the four countries discussed. The second answer relates to the efficacy of the BCG vaccine. Scientists were uncertain with respect to the degree of immunity that the vaccine conferred. The third answer probably relates to the postcolonial state’s faith in the proverbial magic bullet, i.e. the BCG, while overlooking the underlying causes of TB such as under-nutrition and poverty, given the paucity of financial resources. The main purpose of this article is not to blame historical agents for shortcomings in TB control. Rather, I seek to understand administrative fractures within international health and the postcolonial state in South and Southeast Asia of the 1950s which have impeded control efforts to the present.

The common denominator underlying TB control in postcolonial South India and Southeast Asia during the 1950s was the notion of fractured sovereignties. One interesting cleavage in international health was the disagreement between the WHO and the UNICEF regarding the deployment of BCG vaccination in mass campaigns. Whereas the WHO expressed uncertainty in 1948— with respect to large-scale deployment of BCG—the UNICEF advocated mass vaccinations, given the high incidence of TB among children in war-ravaged Europe. A second interesting fault line was conspicuous in the state of Madras, South India when mass vaccination against TB was introduced in 1949. Raman’s articles in *People’s Health* highlight the critique within educated Indian circles of using the notional magic bullet approach in TB control while papering over the underlying social causes of disease. Raman critiqued BCG as a “cheap panacea” to the TB problem, compared to the much more interventionist approach of environmental hygiene ^143^. In contrast, Rajaji’s opposition to the vaccine, subsequent to his resignation as Chief Minister of Madras state was linked to the centralising tendencies of Delhi. The early opposition to BCG in South India, spearheaded by Raman (1948–1951) was hardly unique. Philippine physicians, especially Penaflor were equally sceptical of the government’s advocacy of BCG as opposed to improving people’s living standards. A third discerning fracture that impeded TB control across each of the four countries discussed in the study was administrative ^144^. Public health was a provincial subject in India, Indonesia, the Philippines, and Burma. As a result, financing particular aspects of TB control, especially administration of treatment to individual patients were delegated to provinces whereas the central government was in-charge of formulating policy directives. There was no uniform official or civilian response to policies prescribed by the central government or international agencies ^145^. The process of creating administrative consensus within the postcolonial state while implementing various aspects of TB control was complex and necessitated varying levels of central and local government involvement. This assessment is borne out by troubled expansion of TB control in each of the four countries. Apart from scepticism within India, Indonesia, and the Philippines regarding the efficacy of BCG, the prescriptions of WHO were neither universally accepted nor welcomed. In Burma for instance, the SEARO faced considerable delays in securing permission from the Ministry of Health in conducting a country-wide study assessing the prevalence of TB. Nationalist concerns related to desire for self-sufficiency partly fed Burmese reservations regarding SEARO assistance.

By the 1960s, TB no longer dominated the headlines of global newspapers because its prevalence declined across Europe and North America due to improved living standards. Consequently, the US government cut funding for TB research by the late 1960s ^146^. During the 1970s, the prevalence of TB diverged along the fault lines of the global economy, with its prevalence becoming rare in advanced countries ^147^. In 1978, with the global eradication of smallpox on the horizon and the Alma Ata Declaration on Primary Healthcare, the future for eradication of infectious diseases seemed promising. But, national governments and international aid agencies were slow to contribute resources to the ambitious Alma Ata agenda on Primary Healthcare and embraced the notion of selective primary healthcare. TB and leprosy were eliminated from the program of selective primary healthcare as these diseases were deemed too costly and time-consuming to treat. But, by the late 1980s, the HIV/AIDS pandemic in sub-Saharan Africa produced noticeable increase in TB notifications from the region. The disintegration of the USSR in 1991 was marked by socio-economic crisis and collapse of the health system which led to increased incidence of TB in that part of the world. By 1993, the World Bank began to use the notion of Disability Adjusted Life Years (DALY) to measure the cost-effectiveness of health interventions. As a result of this new economic calculus, the WHO promoted short-course chemotherapy for TB, or Directly Observed Treatment Short Course (DOTS) ^148^. The DOTS subscribed to the notion of selective primary healthcare: simple to treat, inexpensive, use of smear microscopy alone to detect tuberculosis, and directly supervising treatment of individual patients. But, DOTS was not successful in detecting drug-resistant strains of tuberculosis.

According to the WHO Global TB Report (2017), five countries: India, Indonesia, China, the Philippines, and Pakistan contributed to over 50% of TB cases globally ^149^. In 2016, of the 600,000 reported cases of drug-resistant TB, 47% were from India, China, and Russia ^150^. The WHO’s End TB Strategy, set for 2020, targets 35% reduction in TB deaths and a 20% reduction in TB incidence, compared with 2015 levels ^151^. The principles of the End TB Strategy include patient-centred care and prevention (including testing patients for possible drug-resistance strains); bold policies (including redressing socio-economic imbalances); and intensified research and innovation ^152^. Despite India, Indonesia, and the Philippines registering annual economic growth of over 5% in an otherwise sluggish global economy, out-of-pocket expenditures on healthcare accounted for at least 45% of total health expenditures for households ^153^. According to the WHO, there has been a widespread underreporting of TB cases from India and Indonesia due to poor geographical and financial access to healthcare which delays treatment ^154^. The private health sector diagnoses and treats approximately two-thirds of TB patients in India ^155^. There have been cases of private practitioners misdiagnosing TB as silicosis as the latter is compensable ^156^. Private practitioners often prescribe antibiotics to patients without the need for them to undergo the sputum test ^157^. In the absence of strict implementation laws, there are gaps in reporting TB cases treated by private practitioners in India.

In India, the Central Tuberculosis Division proposed a budget of US$ 881 million (2012–2017) to ensure universal treatment access for TB patients under the Revised National Tuberculosis Control Program (RNTCP) ^158^. However the Central Tuberculosis Division was allocated only US$ 680 million ^159^. There are deep-seated funding issues in the management of RNTCP. Currently, the RNTCP is under the Central Tuberculosis Division, a part of the Ministry of Health and Family Welfare. Financial management of state-level and district-level TB control programs are devolved to the local administration. Fund flows from the central government to state or district level TB control programs can be problematic which has delayed wage payment to contractual staff employed with the RNTCP ^160^.

Since 1995, DOTS has become the cornerstone of TB control in Indonesia. The *Puskesmas* (Community Health Centres) have formed the nucleus of TB detection and treatment. In 1999, following the Asian monetary crisis (1997–98), Indonesia embarked on a program of administrative decentralisation. Beginning 2001, healthcare has been delegated to the districts, with block grants from the central government ^161^. Funding for disease control have not been prioritised by local governments due to resource deficits ^162^. Since 2000, Indonesia has received financial assistance from various international sources, particularly the Global Fund to Fight AIDS, Tuberculosis, and Malaria, USAID (United States Agency for International Development), and others to compensate the local governments’ deficit spending on health. The Global Fund disbursed grants at the district level and funded the operations of the country’s DOTS program. As a result of effective case finding, TB case notifications registered a steep increase from 44.5 in 2001 to 119 in 2007 and case detection increased from 30% to over 76% ^163^. 90% of detected cases were successfully treated. By 2006, Indonesia became the first country in the SEARO region to meet the benchmarks for TB case detection and cure. But, with the suspension of Global Fund in 2007, successes registered by the Indonesian DOTS program during first decade of the twenty-first century have been reversed due to cutbacks on health manpower and there has been a decrease in TB case finding.

As in the case of Indonesia, TB control in the Philippines is devolved to the Rural Health Units whereas the National Center for Disease Control formulates guidelines on TB control. The implementation of the DOTS program, introduced in 1997, is devolved to the Rural Health Units in the decentralised administrative set-up. According to the 2011 Philippine Department of Health estimate although the DOTS program has been successful in treating at least 91% of TB patients, the Philippine Plan to Control TB between 2010 and 2016 (PhilPACT) faces several logistical challenges ^164^. The Philippine Department of Health estimates that at least 57.2% of patients are treated by private practitioners ^165^. Since 2008—as TB was removed from the Department of Health’s list of notifiable diseases—there has been a serious under-enumeration of the prevalence of TB in the Philippines. This limits the ability of the Philippine Department of Health to ensure notifications of the disease from private practitioners. Private practitioners sell anti-TB drugs over-the-counter. The high cost of anti-TB drugs may be a barrier for patients in continuing treatment. Filipino private practitioners rely on X-ray findings only and not on sputum smear microscopy in detecting TB. As a result, there is a possibility of over-diagnosis or under-diagnosis of the disease which exposes patients to inappropriate drug regimens and the risk of contracting drug-resistant TB ^166^. Stigma associated with diagnosis, misconceptions regarding TB (for e.g. the disease is spread through sharing utensils, alcoholism, or strenuous physical activity), or the perceived high cost of TB care are some of the contributory factors delaying timely detection and treatment of the disease ^167^. At least 30% of detected Multi-Drug Resistant TB (MDR) patients are unable to complete their treatment regimens due to the long duration of treatment, and adverse side-effects of medication ^168^.

Myanmar (formerly Burma) introduced DOTS as a component of the National TB program in 2003. The SEARO estimates that Myanmar has the highest incidence of TB in the region (estimated at 381 per 100,000 population in 2011) ^169^. 1.5% of the nation’s population is annually affected with TB and approximately 85,000 people progress to contract the disease ^170^. Tuberculosis case fatalities in Myanmar are among the highest in the region due to high mortality among patients exhibiting HIV TB co-infection ^171^. The Ministry of Health has devolved administration of the National Tuberculosis program of the country to Township Health Departments (THD) which carry out routine disease control activities, particularly detecting and treating TB cases. Although TB diagnostic services are in principle available at the THD, patients often have to travel to district-level TB centres for sputum examinations. The choice influencing TB patients’ treatment with either the General Practitioner (GP) or THD is contingent upon a variety of factors such as practitioners’ ability to provide symptomatic relief, financial constraints, or accessibility of the treatment centre. Nearly three-quarters of Myanmar’s TB patients initially consult with their GPs, irrespective of socio-economic status, given the latter’s ability to provide individualised care and turn to the THD only for receiving the anti-TB medicines provided free-of-charge ^172^. The largely-unregulated private sector treats the majority of TB patients in Myanmar. TB cases, even if diagnosed by GPs, are not notified in accordance with the provisions of the country’s National Tuberculosis Program ^173^. Major constraints with respect to TB control in Myanmar include limited reach of the National Tuberculosis program in conflict-prone areas and treatment of only 2% of diagnosed MDR patients ^174^. The problem of MDR TB in Myanmar has been compounded by stock-outs of anti-TB drugs, sale of counterfeit drugs in private pharmacies, and self-treatment, or discontinuation of therapy by TB patients due to financial difficulties.

During the 1990s, many countries in South and Southeast Asia underwent a period of health reform to address issues appertaining to accessibility and efficiency. But, weak regulatory mechanisms outlining the role of private practitioners in National TB Control Programs in India, Indonesia, the Philippines, and Burma, and unclear lines of demarcation between provincial and central governments with respect to management of the DOTS program have hampered global control efforts. The global StopTB Strategy (2001–2005) sought to expand DOTS and redress challenges of HIV and MDR TB ^175^. Between 1995 and 2008, DOTS was successful in absolute numbers with respect to averting approximately 6 million TB deaths and curing 36 million patients ^176^. But, weak regulatory mechanisms governing the role of private sector participation in disease control have prevented timely reporting of detected TB cases to health authorities.

This study reveals the benefits of linking the history of tuberculosis in Southern India and Southeast Asia to the underlying tensions in international health of the 1950s between a holistic approach that correlated disease to poverty and a narrow technocentric approach that focused on targeted interventions such as vaccinations. At the time, in each of the countries discussed in the study, there was a growing awareness of the general public with respect to the economic and social underpinnings of TB. Yet, governments turned to magic bullets in the hope that they could paper over underlying socio-economic causes of ill health. If I were to pick a dominant theme from the paper, it would be one about how the discovery of wonder drugs and BCG, concomitant with the independence of India, Indonesia, the Philippines and Burma ignited new hopes for TB control by 1950. Yet, these hopes have continued to elude planners from the 1950s to the present day due to a dualism between health policy formulation at the centre and implementation at the level of local governments. These dualisms have made it difficult for TB control to be integrated within the overall framework of public health activities of the postcolonial state.

## Data availability

All data underlying the results are available as part of the article and no additional source data are required.

## Footnotes

^1^WHO, *Global Tuberculosis Report 2017* (Geneva: WHO, 2017), 1.

^2^Ibid.

^3^Christian McMillen *Discovering Tuberculosis: A Global History, 1900 to the Present* (New Haven: Yale University Press, 2015), 121.

^4^P.S Chandrashekhar, *Consumption in Madras* (Madras: Indian Association, 1914), Shelfmark M9790, Wellcome Library Closed Stores.

^5^Ibid.

^6^Helen Bynum, “Tuberculosis: An Ancient and Deadly Foe,” in *Tuberculosis: A Short History*, Alexander Medcalf, Henrice Altink, Sanjoy Bhattacharya, and Monica Saavedra, eds. (Hyderabad: Orient Blackswan, 2013), 1–2.

^7^Niels Brimnes, “The Global Tuberculosis Program of the WHO,” in *Tuberculosis: A Short History*, Alexander Medcalf, Henrice Altink, Sanjoy Bhattacharya, and Monica Saavedra, eds. (Hyderabad: Orient Blackswan, 2013), 19.

^8^“Excerpts of an Interview of Milton Siegel with John Charnow, 7 December 1984,” Doc. No. CF RAI/USAA/DB01/HS/1996-0061, UNICEF Archives.

^9^Ibid.

^10^UNICEF, “UNICEF Program Committee: Report on Progress of the BCG Program, 19 January 1949,” Doc E/ICEF/94, *United Nations Economic and Social Council*, UN Archives.

^11^Linda Bryder, “We Shall Not Find Salvation: BCG Vaccination in Scandinavia, Britain, and USA,” *Social Science and Medicine* (1999): 1157–67.

^12^Bryder, “We Shall Not Find Salvation,” 1162.

^13^Joseph Aronson and Caroll Palmer, “Experience with the BCG Vaccine in the Control of Tuberculosis among North American Indians,” *Public Health Reports* no. 61 (1946): 802–20.

^14^Aronson and Palmer, “Experiences with the BCG Vaccine,” 820.

^15^Ibid.

^16^Ibid.

^17^Editorial, “BCG,” *American Journal of Public Health* 37, no. 3 (1947): 307–9.

^18^“Results of Recent International Studies on Tuberculosis,” *Chronicle of the World Health Organization* 9 (1955), WHO Library (WHOL).

^19^“First Results of Mass Campaign with BCG,” *Chronicle of the World Health Organization* 4 (1950), WHOL.

^20^Bryder, “We Shall Not Find Salvation,” 1164.

^21^Bryder, “We Shall Not Find Salvation,” 1162.

^22^On the international aspects of the tuberculosis campaign in South India see for example Niels Brimnes, “BCG Vaccination and WHO’s Global Strategy of Tuberculosis Control, 1948–83,” *Social Science and Medicine* 67 (2008): 863–73; Christian Mcmillen and Niels Brimnes, “Medical Modernisation and Medical Nationalism: Resistance to Mass Tuberculosis Vaccination in Post-Colonial India,” *Comparative Studies in Society and History* 52, no.1 (2010): 180–209. For a nuanced understanding of how Sri Lanka represents an anomaly with respect to internationally-financed healthcare in South Asia, refer Margaret Jones, “Policy Control and Pathways: Tuberculosis Control in Sri Lanka, 1948–1990,” *Medical History* 6, no.4 (2016): 514–33. In her article, Jones describes how Sri Lanka in the mid-1960s sought to supplant its earlier successful model of TB control of the 1950s—based on a combination of BCG vaccination, case-detection, and treatment— with a WHO-influenced community healthcare model adapted from India. The latter sought to integrate TB into routine activities of the local health centres to minimise costs but failed. Jones’ article highlights the limitations encountered in grafting the WHO’s model of TB control onto the existing health system already operating in Sri Lanka.

^23^For a rare exception see Sunil Amrith, “In Search of a Magic Bullet for Tuberculosis: South India and Beyond, 1955–65,” *Social History of Medicine* 17, no. 1 (2004):113–30. Amrith’s article examines social and economic factors influencing compliance of individual patients with anti-tuberculosis drugs in South India.

^24^Randall Packard, *White Plague, Black Labor: Tuberculosis and the Political Economy of Health and Disease in South Africa* (Berkeley: University of California Press, 1989).

^25^For the most recent study documenting TB control in international health contexts refer McMillen, *Discovering Tuberculosis*. See also Niels Brimnes, *Languished Hopes: Tuberculosis, the State and International Assistance in Twentieth Century India* (Hyderabad: Orient Blackswan, 2016), 280–81.

^26^Brimnes, *Languished Hopes*, 280–84.

^27^Brimnes, *Languished Hopes*, 156. At the time, the then Madras Chief Minister C. Rajagopalachari, also known as Rajaji (1952–54), a veteran nationalist leader of the Congress Party had an uncertain term in office as he faced political opposition from the Dravidian movement and its anti-Brahmin rhetoric for promoting Hindi as the national language during the 1930s. Rajaji’s resistance to BCG was earlier spearheaded by A.V. Raman, a retired sanitary engineer from Madras and later the editor of *People’s Health*, between 1946 and 1951. He was deeply influenced by Mahatma Gandhi’s idea of rural sanitation.

^28^With reference to postcolonial Southeast Asia, this study focuses exclusively on Indonesia, the Philippines and Burma, countries which had gained independence from colonial rule prior to 1950.

^29^ *Purdah* refers to the social practice in South Asian Hindu and Muslim households of screening women from men by means of a curtain.

^30^Arthur Lankester, *Tuberculosis in India: Its Prevalence, Causation and Distribution* (Calcutta: Butterworth and Co, 1920), 46.

^31^Ibid.

^32^S.K. Mullick, *Consumption in Calcutta* (Calcutta: United Press, 1918), Shelfmark Asia, Pacific, and Africa T35032(f), BLL01012507118, British Library.

^33^TB Association of India, *Transactions of the Tuberculosis Workers Conference Held in New Delhi, November 1939, Under the Auspices of the Tuberculosis Association of India* (New Delhi: Tuberculosis Association of India, 1939), Shelfmark 31009, Wellcome Library Closed Stores Medical.

^34^Editorial, “Fight the Big Three,” *The Antiseptic* 42, no. 8 (1945): 457–60.

^35^Editorial, “Fight the Big Three,” 457.

^36^Health was a provincial subject since 1919, in accordance with the Government of India Act (1919). An inadvertent consequence of transferring health to provincial government was increased politicisation. Disease control campaigns had to operate under the constraints of politicised provincial administration with limited funding. For details, see William Summers, “Cholera and Plague in India: The Bacteriophage Inquiry of 1927–1936,” *Journal of the History of Medicine and Allied Sciences* 48 (1993): 275–301, 282.

^37^“First Things First in Tuberculosis Campaign in India: What Should be in the Program for Five Years,” *Transactions of the Fourth Tuberculosis Workers’ Conference Held in New Delhi* (New Delhi: The Tuberculosis Association of India, 1946), 5.

^38^Ibid.

^39^Ibid.

^40^K. Vasudeva Rao, “Factors Influencing the Spread of Tuberculosis and Measures to be Taken to Combat the Same,” *The Miscellany: Journal of the South Indian Branch of the Indian Medical Association* 14, no. 4(1947): 1–5.

^41^Rao, “Factors Influencing the Spread of Tuberculosis,” 2.

^42^Tuberculosis Association of India, *Proceedings of the Sixth Tuberculosis Workers’ Conference Held in Calcutta, December 1948, Under the Auspices of the Tuberculosis Association of India* (New Delhi: Tuberculosis Association of India, 1949), Shelfmark K31014, Wellcome Library Closed Stores Medical.

^43^J.B. MacDougall, “A Survey of Tuberculosis Control Measures in Burma, Ceylon, and Certain Parts of India with Conclusions and Recommendations,” 3 January 1950, Registry File DC TB SEARO, WHO Archives (WHOA).

^44^Ibid.

^45^Ibid.

^46^Ibid.

^47^His Excellency The Maharajah of Bhavanagar (The Governor of Madras), “BCG Vaccination: Madras 15-2-1949,” *The Miscellany: Monthly Journal of the South Indian Branch of the Indian Medical Association* 15, no.8 (1949): 2–3.

^48^The Maharajah of Bhavanagar, “BCG Vaccination,” 3.

^49^“Letter from Rajkumari Amrit Kaur to T.S.S. Rajan,” 18 September 1949, GO 1313 *Health Department*, M.S. Series (15 April 1950), Tamil Nadu Archives (TNA).

^50^Ibid.

^51^McMillen, *Discovering Tuberculosis*, 272.

^52^“Draft of Letter from T.S.S. Rajan to P.V. Benjamin on Tuberculosis Vaccination, “14 September 1949, G.O. 1313 *Health Department*, M.S. Series (15 April 1950), TNA.

^53^Ibid.

^54^A.V. Raman, “BCG, What About It,” *People’s Health* 3, no. 3 (1949).

^55^A.B. Shetty, “Address Delivered by the Honourable A.B. Shetty, Minister of Public Health Government of Madras at the Inauguration of the BCG Campaign on 15 February 1949 at Madras Medical College,” *The Miscellany: Monthly Journal of the South Indian Branch of the Indian Medical Association* 15, no. 8 (1949) 2–4.

^56^Shetty, “Address,” 3. The Zamindari system refers to a land-tenure system extant in pre-independent India.

^57^Ibid.

^58^Lionel Dole, “French Doctors Doubt Efficacy of BCG,” *People’s Health* 4, no.4 (1950): 149–52.

^59^Ibid.

^60^Dole, “French Doctors,” 150.

^61^A.V. Raman, “Editorial,” *People’s Health* 4, no.4 (1950): 177–80.

^62^A.V. Raman, “American Citizens’ View on BCG,” *People’s Health* 4, no. 4 (1950): 248–50.

^63^A.V. Raman, “Editorial: A Precious Child but Born in a Bad Environment,” *People’s Health* 4, no. 1 (1950): 134.

^64^For episodes appertaining to resistance in centrally-driven vaccination campaigns, see for example Vivek Neelakantan, “Eradicating Smallpox in Indonesia: The Archipelagic Challenge,” *Health and History* 12, no. 1 (2010): 61–87.

^65^For a detailed understanding of Nehruvian Science refer David Arnold, “Nehruvian Science and Postcolonial India,” *ISIS* 104, no. 2 (2013): 360–70, 365.

^66^“Letter from the Madras Health Minister to Rajkumari Amrit Kaur,” 12 July 1955, GO 3186 MS Series (12 October 1955), *Health Department*, TNA.

^67^C. Rajagopalachari, *BCG Vaccination: Why I Oppose It* (Madras: Jupiter Press, 1955), 7, Accession No. 109290, Roja Muthiah Research Library.

^68^Rajagopalachari, *BCG Vaccination*, 3.

^69^“Tuberculosis Vaccination, Issue of a Booklet Truth About BCG: Why the Government has Launched a Mass Campaign,” 26 August 1955, GO 2682 M.S. Series, TNA.

^70^“Tuberculosis: BCG Vaccination in Cuddalore, Alleged Complications: Press Note Issued by Government,” 15 September 1955, G.O. 3069 M.S. Series, TNA.

^71^Rajagopalachari, “BCG Vaccination,” 35.

^72^“Tuberculosis Vaccination, Issue of a Booklet Truth About BCG.”

^73^Ibid.

^74^Elizabeth Fee, Marcos Cueto, and Theodore Brown, “At the Roots of the World Health Organization’s Challenges: Politics and Regionalization,” *American Journal of Public Health* 106, no. 11 (2016): 1912–17.

^75^Ibid. Between 1948 and 1951, the WHO established regional offices in the Americas (PAHO), Southeast Asia (SEARO), Europe (EURO), Eastern Mediterranean (EMRO), the Western Pacific (WPRO), and Africa (AFRO). SEARO member states at the time included India, Thailand, Indonesia, Sri Lanka, Burma, Afghanistan, and Thailand. However, owing to the partition of British India and the tensions over Kashmir, Pakistan acceded to the EMRO. Indonesia’s accession to the SEARO was complicated due to the Dutch Military Action against the Indonesian Republic in 1948. At the time, the Department of Health of the Netherlands Indies (Departmen van Gezondheid) expressed a serious reservation that the birth of the SEARO would be of little benefit to Indonesia as Batavia (Jakarta) was much closer to the proposed WPRO headquarters at Manila (that would include the Philippines, Vietnam, Cambodia, Laos, Formosa, Japan, Australia, New Zealand, and South Korea) than New Delhi (the SEARO headquarters). At the behest of Jawaharlal Nehru, the republican government (functioning from Yogyakarta) was co-opted into the SEARO, a move that aroused suspicion of the colonial authorities. Subsequent to the transfer of political sovereignty to the republicans in December 1949, Indonesia acceded to the SEARO in order to cement cooperation with India ion international health. Refer Vivek Neelakantan, *Science, Public Health and Nation-Building in Soekarno-Era Indonesia* (Newcastle-upon-Tyne: Cambridge Scholars Publishing, 2017), 68.

^76^Chandra Mani, “International Health: Application of WHO Programs and Policies in a Region,” *American Journal of Public Health,* no. 41 (1951): 1469–72.

^77^Ibid. By 1951, Mani estimated that India recorded approximately 2.5 million new TB cases whereas Java and Madura recorded 750,000 cases for the same year.

^78^SEARO, *Sixty Years of WHO in Southeast Asia: Highlights, 1948–2008* (New Delhi: WHO Regional Office for Southeast Asia), 76. National pilot demonstration projects in the context of tuberculosis control served as laboratories for developing countries to bridge the gap between available epidemiological knowledge derived from clinical research, on one hand and application of such knowledge to local conditions, on the other.

^79^J.C. Tao, *Tuberculosis Control in the West Pacific Region, 1951–1970* (Manila: WPRO, 1972), WHO Global Digital Library, accessed 1 March 2018, http://iris.wpro.who.int/handle/10665.1/6828.

^80^“Report of the Study Group on Tuberculosis during Technical Discussions: 6 ^th^ World Health Assembly,” 19 May 1953, Doc. A6/Technical Discussions/Tuberculosis/19, WHO Global Digital Library, accessed 1 March 2018, http://apps.who.int/iris/bitstream/10665/102980/1/WHA6_TD-Tuberculosis-19_eng.pdf.

^81^Johannes Leimena, “Ten Year Activities of the Ministry of Health,” *Berita Kementerian Kesehatan* 5, no. 2 (1956): 5–12.

^82^Neelakantan, *Science, Public Health, and Nation-Building*, 1.

^83^L.G.J. Samallo, “Lima Tahun Usaha Pemberantasan Penjakit Tuberculose di Jogjakarta,” *Berita Kementerian Kesehatan Republik Indonesia* 3, no. 5 (1956): 46–53.

^84^Samallo, 51.

^85^K. Osterkov Jensen, “Assignment Report on Tuberculosis Control in Indonesia,” 25 April 1958, WHO Project Indonesia 8 B, Doc. SEA TB/5, SEARO Library (New Delhi: SEARO, 1958).

^86^“Pidato Pembukaan Drs. L.G.J. Samallo, Kepala Bagian Pemberantasan Penjakit Tuberculosa: Conferentie Pemberantasan Penjakit Tuberculose di Bandung,” 8–13 August 1955, *Berita Tuberculosea Indonesiansis* (Jogjakarta: Kementerian Kesehatan, 1955), 6–11.

^87^C.F. Borchgrevink and Nio Kok Hien, “Report on the BCG Campaign in Indonesia,” November 1953-March 1956, Doc SEA/TB/4, *WHO Project Indonesia 8A*, *Project Files*, WHOA [Restricted].

^88^L.G.J. Samallo, “Penilaian Hasil Hasil Vaksinasi BCG Besar Besaran di Indonesia,” In *Laporan Kongres Ilmu Pengetahuan Nasional Pertama*, vol. 1 MIPI, ed. (Djakarta: Kementerian Penerangan, 1958).

^89^Ibid.

^90^Ibid.

^91^Borchgrevink and Hien, “BCG Campaign.”

^92^Ibid.

^93^Neelakantan, “Eradicating Smallpox,” 71.

^94^Ibid. Smallpox cases in Makassar during the 1956 outbreak were often isolated in a leprosy ward.

^95^According to the Chinese medical cosmology, diseases are cured by restoring the natural balance between the complementary opposing forces, *yin* and *yang*. The BCG vaccine disrupted the body’s natural process of healing.

^96^Eugenio Alonso, “44th Philippine Medical Association and the First Southeast Asia Medical Conference, Manila,” *The Philippine Medical World* (1956):n.p.

^97^Ibid.

^98^C. Penaflor, “The Post-World War Tuberculosis Problem in the Philippines,” *Diseases of the Chest* 26, no. 1 (1954): 52–61.

^99^Ibid.

^100^Ibid.

^101^Ibid.

^102^Penaflor, “Tuberculosis Problem,” 54.

^103^Penaflor, “Tuberculosis Problem,” 55.

^104^Penaflor, “Tuberculosis Problem,” 57.

^105^F Van Deinse, “Vaccination against Tuberculosis with Freeze-Dried BCG Vaccine,” *American Journal of Public Health* 41, no. 10 (1951): 1209–14.

^106^Sofin Bona De Santos, Francisco Dungo, and Alejandro Aaonan, “Experiences with Dry BCG Vaccine in the Philippines,” *Diseases of the Chest* 19, no. 5 (1951): 544–65.

^107^M.B. Abad and M. Elicano, “Tuberculosis among BCG’ed Children,” *Journal of the Philippine Islands Medical Association* 31, no. 10 (1955): 552–60.

^108^“Memorandum from Anton Geser to I.C. Fang WHO Regional Committee for the West Pacific (WPRO) Regional Director: Narrative Report for October 1952,” File DC-TB, *WHO Registry Files*, WHOA.

^109^Ibid.

^110^A.A. Angara and D.R. Huggins, “Report on Field Visit to Ilocos Norte, Ilocos Sur, Pangasinan, and Bulacan,” 31 December 1959, Doc. WPR/PHA/FR/7, File Philippines 17, WPRO Library.

^111^Vivek Neelakantan *,* “Disease Eradication and National Reconstruction in Indonesia and the Philippines during the Early Decades of the Cold War,” *IIAS Newsletter* 71 (2015): 4–5.

^112^“Evaluation Report of the BCG Mass Vaccination Project in the Philippines, 1952–56,” File Philippines 17, *Project Files*, WHOA.

^113^Angara and Huggins, “Report on Field Visit.”

^114^A.A. Angara, “BCG Vaccination and Provincial Health Services: A Special Report,” September 1959, *Project Files*, WHOA.

^115^Angara and Huggins, “Report on Field Visit.”

^116^Angara and Huggins, “Report on Field Visit.”

^117^Ibid.

^118^Angara, “BCG Vaccination.”

^119^M.B. Abad, N. Cortes, and L.A. Vasquez, “Further Observations on Clinical Tuberculosis among BCG’ed Filipino Children,” *Journal of the Philippine Islands Medical Association* 37, no. 11 (1961): 837–43.

^120^Ibid.

^121^Ibid.

^122^Ibid.

^123^Neelakantan, “Disease Eradication and National Reconstruction.”

^124^Ibid.

^125^WPRO, “Annual Report of the Regional Director for 1961,” Doc. WP/RC 12/2, *WPRO Historical Collections* (Manila: WPRO, 1961).

^126^SEARO, “Third Annual Report of the Regional Director to the Regional Committee for Southeast Asia,” Doc. SEA/RC4/2, *SEARO Historical Collections* (New Delhi: SEARO, 1951).

^127^Hugh Tinker, *The Union of Burma: A Study of the First Years of Independence* (Oxford: Oxford University Press, 1957).

^128^Tharapi Than, “The Languages of Pyidawtha and Burmese Approaches to National Development,” *Southeast Asia Research* 21, no. 4 (2013): 639–54.

^129^Than, “The Languages of Pyidawtha,” 646.

^130^Ibid.

^131^Tinker, *The Union of Burma*, 215–16.

^132^Tinker, *The Union of Burma*, 217.

^133^SEARO, “Fifth Annual Report of the Regional Director to the Regional Committee for Southeast Asia,” Doc. SEA/RC6/2, *SEARO Historical Collections* (New Delhi: SEARO, 1953).

^134^Review, “Eight Months of Anti-Tuberculosis Work in Burma,” *Tubercle* (1953): 28–29.

^135^Ibid.

^136^SEARO, “Fifth Annual Report.”

^137^Review, “Eight Months of Anti-Tuberculosis Work.” For understanding the Burmese cosmology of disease, see also Monique Skidmore, “Contemporary Medical Pluralism in Burma,” in *Dictatorship, Disorder, and Decline in Myanmar*, Monique Skidmore and Trevor Wilson, eds., 193–209 (Canberra: ANU E-Press, 2007).

^138^SEARO, “Fifth Annual Report.”

^139^SEARO, “Ninth Annual Report of the Regional Director to the Regional Committee for Southeast Asia,” Doc. SEA/RC10/2, *SEARO Historical Collections* (New Delhi: SEARO, 1957).

^140^SEARO, “Eighth Annual Report of the Regional Director to the Regional Committee for Southeast Asia,” Doc. SEA/RC9/2, *SEARO Historical Collections* (New Delhi: SEARO, 1956).

^141^“SEARO Regional Director Meeting with Area Representatives: Summary of Minutes,” Doc. SEA/AR3/2 (New Delhi: SEARO, 1955), WHOL.

^142^Pramoedya Ananta Toer, “My Kampung,” *Indonesia* 61 (1996): 25–32.

^143^Brimnes, *Languished Hopes*, 170–74.

^144^For administrative fractures that impeded the functioning of the Ministry of Health in an Indian context, see Shirish Kavadi, “Medicine, Philanthropy and Nationhood: Tensions of Different Visions in India,” in *Public Health and National Reconstruction in Post-War Asia: International Influences: Local Transformations*, Liping Bu and Ka-che Yip, eds., 132–53 (Abingdon: Routledge, 2014).

^145^See, for example, Sanjoy Bhattacharya, *Expunging Variola: The Control and Eradication of Smallpox in India, 1947–1977* (Hyderabad: Orient Longman, 2005).

^146^Salman Keshavjee and Paul Farmer, “Tuberculosis, Drug Resistance, and the History of Modern Medicine,” *New England Journal of Medicine* 367 (2012): 931–6.

^147^Ibid.

^148^Ibid.

^149^WHO, *Global Tuberculosis Report 2017* (Geneva: WHO, 2017), 1.

^150^Ibid.

^151^WHO, *Global Tuberculosis Report*, 2.

^152^WHO, *Global Tuberculosis Report*, 11.

^153^WHO, *Global Tuberculosis Report*, 126.

^154^WHO, *Global Tuberculosis Report*, 78.

^155^EPW Editorial, “At the Brunt of Tuberculosis,” *Economic and Political Weekly* LI, no. 42 (2016): 7–8.

^156^Ibid.

^157^Ibid.

^158^Ranjith Babu, Karuna Sagili, Anil Jacob, and Sarabjit Chadha, “Resource Optimisation for Tuberculosis Elimination in India,” *Economic and Political Weekly* LI, no. 19 (2016): 26–27.

^159^Ibid.

^160^Ibid.

^161^WHO, *A Brief History of Tuberculosis Control in Indonesia* (Geneva: WHO, 2009).

^162^Ibid.

^163^Ibid.

^164^Department of Health, *National Tuberculosis Control Program* (Manila: Department of Health, 2014), The Philippine Department of Health Publications Website, accessed 8 April 2018 http://www.doh.gov.ph/sites/default/files/publications/Updated_PhilPACT_2013-2016_v080715.pdf.

^165^J.L. Portero and M. Rubio, “Private Practitioners and Tuberculosis Control in the Philippines: Strangers When They Meet?,” *Tropical Medicine and International Health* 8, no. 4 (2003): 329–35.

^166^Ibid.

^167^Department of Health, *National Tuberculosis Control Program*. For a nuanced understanding of tuberculosis-associated stigma in the Philippines, see Filomeno Aguilar Jr., “Targeting Tuberculars: Social Stigma and Public Health Campaigns,” *Philippine* Studies 57, no.2 (2009): 293–306. In Philippine popular culture, the aetiology of tuberculosis is not well-understood. Filipinos think of TB in terms of predisposing factors such as lack of food, overwork and exposure to the elements, especially letting sweat dry on one’s back, which weaken the body and disrupt the hot-cold balance. Heredity is perceived as an important factor (for e.g. TB is transmitted through the blood of one’s parents). Given these contradictory perceptions, a tuberculosis patient (“tubercular”) is seen as personally responsible for contracting the disease and is stigmatised. Under the weight of stigma, patients enrolled for DOTS treatment and their immediate family members will conceal the disease, risking the transmission of TB to contacts. Case statistics in official DOTS reports do not capture missing TB patients.

^168^Department of Health, *Joint Tuberculosis Program Review: Philippines, 2013* (Manila: Department of Health, 2014).

^169^SEARO, *Tuberculosis Control in the South-East Asia Region: Annual TB Report 2013* (New Delhi: SEARO, 2013), SEARO Website, accessed 9 April 2018, http://www.searo.who.int/tb/annual_tb_report_2013.pdf.

^170^Saw Saw *et al.*, “Public and/or Private Healthcare: Tuberculosis Patients’ Perspective in Myanmar,” *Health Research Policy and Systems* 19, no.7 (2009), https://doi.org/10.1186/1478-4505-7-19.

^171^SEARO, *Tuberculosis Control*.

^172^Ibid.

^173^For details refer Knut Lönnroth *et al.*, “Social Franchising of TB Care through Private GPs in Myanmar: An Assessment of Treatment Results, Access, Equity, and Financial Protection,” *Health Policy and Planning* 22 (2007): 156–66.

^174^WHO Country Office for Myanmar, “Multidrug-Resistant Tuberculosis in Myanmar: Programs Plans and Challenges: Factsheet,” SEARO Media Centre Website, accessed 10 April 2018, http://www.searo.who.int/myanmar/mediacentre/MDT-TB_Factsheet.pdf.

^175^Rifat Atun, Diana Weil, Mao Tan Eang, and David Mwakyusa, “Health-System Strengthening and Tuberculosis Control,” *The Lancet* 375, no. 9732 (2010): 2169–78.

^176^Ibid.

