# Supplementary material for: Tuberculosis control in postcolonial South India and Southeast Asia: Fractured sovereignties in international health, 1948-1960
Source: Wellcome Open Res. 2018 Apr 17;2:4. Originally published 2017 Jan 13. [Version 2] doi: 10.12688/wellcomeopenres.10544.2 (PMC5920540; doi:10.12688/wellcomeopenres.10544.2)
Supplement: Peer review history

# Peer review: Referee response for version 2

**DOI:** 10.21956/wellcomeopenres.15843.r32919

**Published:** 2018-05-24

**Authors:** Susan Heydon

**Affiliations:** 1 School of Pharmacy, University of Otago, Dunedin, New Zealand

I am pleased by the way the author has addressed my comments and those of the other reviewers. I believe it is a much stronger but more balanced paper. I am happy for the status to be changed to approved and wish the author well.

I have read this submission. I believe that I have an appropriate level of expertise to confirm that it is of an acceptable scientific standard.

# Peer review: Referee response for version 2

**DOI:** 10.21956/wellcomeopenres.15843.r33003

**Published:** 2018-04-27

**Authors:** Christian W. McMillen

**Affiliations:** 1 Cocoran Department of History, University of Virginia, Charlottesville, VA, USA

I am pleased with the revisions. The author’s attention to my concerns, as well as the other reviewers’ queries, is laudable. I think the status of the paper should be amended to approved.

I have read this submission. I believe that I have an appropriate level of expertise to confirm that it is of an acceptable scientific standard.

# Peer review: Referee response for version 2

**DOI:** 10.21956/wellcomeopenres.15843.r32917

**Published:** 2018-04-25

**Authors:** Liping Bu

**Affiliations:** 1 Department of History, Alma College, Alma, MI, USA

I think Neelakantan V. has well addressed the issues regarding his version.  The article is now good for publication.

I have read this submission. I believe that I have an appropriate level of expertise to confirm that it is of an acceptable scientific standard.

# Peer review: Referee response for version 1

**DOI:** 10.21956/wellcomeopenres.11362.r19309

**Published:** 2017-02-13

**Authors:** George Dehner

**Affiliations:** 1 Department of History, Wichita State University, Wichita, KS, USA

In this article the author sketches out an ambitious, and I agree, important thesis on post-World War II tuberculosis (TB) control and more broadly international health in South and Southeast Asia. Transnational discussion of health programs related to TB control have thus far generally overlooked the political aspects of decolonization in their reviews making this study a welcome addition to the literature on international health. Regrettably, the author is not completely successful in achieving this stated goal.

 The author sets out a difficult task: namely, to examine tuberculosis control and the *Bacillus Calmette-Guerin* (BCG) vaccination programs in India, Burma, Indonesia, and the Philippines. The colonial experience of these states, under the control of the British, Dutch and the United States respectively, differ as does their experiences during the Second World War and their painful and chaotic national births. These states may share common experiences in public health under the colonial regimes and in their early national periods, but the author must do more to highlight these commonalities and not just assume them. 

 Further complicating the author’s self-assigned task is that the vehicle for examining tuberculosis control in these new nations is the UNICEF-funded and WHO-led BCG inoculation programs. The author rightly points out that in this post-war period the WHO emphasized bio-medical technical approaches to disease threats. US financing and diplomatic muscle greatly shaped international health tactics in the 1950s and 1960s and this technical approach mirrored US solutions to public health problems. Narrow-focused technical programs stood in contrast to more holistic frameworks that marked some pre-war League of Nations Health Organization programs and that re-emerged in international health debates at the WHO-sponsored Alma-Ata conference in 1978. But the BCG example is problematic because US public health officials—British health officials as well—were highly skeptical about the vaccine’s effectiveness. While some US health officials were willing to concede that BCG might be effective in protecting children in places where TB was rampant, the major support for the program came from Scandinavian states.  Therefore, a discussion of the contested nature of the BCG vaccine would be appropriate in the background section portion of the introduction.

 The author asserts that health programs were a component of nation-building; a point admirably demonstrated in the India discussion. However, this high standard is not maintained in the examples from the other states of South and Southeast Asia that comprise this study. For example, it would be useful for the author’s argument to know more about the motivations for the 1956 pilot TB program in the Philippines (8). Who were the driving forces in initiating this program—the Filipino government or the United States? Did the WHO have a role? Also, it is not clear from this summary if the program revolved around a BCG distribution system or was there some other model that was being pursued? In similar fashion, it is pertinent to know if the bureaucratic hassles faced by the WHO survey of TB in Burma were part of an assertion of Burmese government independence in matters of health or the result of disputes between central and local authorities (9). Fuller accounts and discussions of these cases would help to bolster the author’s argument.

 The article as written has much to commend it. The section on India is the most fully developed and the one most completely supportive of the author’s conclusions. Expanding the political aspects of TB control, clarifying the unifying elements in their nationalist formation, and tightening up the transitions between sections will go a long way in making this article more effective. In the conclusion, the author states that “resistance to vaccination in postcolonial contexts stems from the association of the vaccine with the colonial state’s interventionist ambitions …” and that there existed a “dualism between health policy formulation at the centre and implementation at the level of local governments” (9-10). These are intriguing assertions, but the article needs to do more to provide evidence that these conclusions are true for nations in the region beyond India.  

I have read this submission. I believe that I have an appropriate level of expertise to confirm that it is of an acceptable scientific standard, however I have significant reservations, as outlined above.

# Response

**Published:** 2018-04-11

**Authors:** Vivek Neelakantan

**Affiliations:** IIT MADRAS, India

**George Dehner, Comment 1**: The BCG example is problematic because US public health officials, British officials as well, were highly sceptical regarding the vaccine’s effectiveness. Elaboration of the contested nature of the BCG vaccine would be appropriate.

**Response: **Contested nature of the BCG vaccine expatiated on page 5, V. 2, in a separate paragraph.

**George  Dehner, Comment 2:** “The author asserts that health programs became a component of nation-building; a point admirably demonstrated in the India discussion. However this high standard is not maintained in the examples of other states of South and Southeast Asia that comprise this study. For example, it would be useful to know more about the motivations of the 1956 pilot TB program in the Philippines:

Who were the driving forces, the WHO or the US?

Did the WHO have a role? It is not clear whether the program revolved around a BCG distribution system or was there some other system being pursued? In a similar fashion it is pertinent to know if the bureaucratic hassles faced by the WHO survey of TB in Burma were part of an assertion of Burmese government independence in matters of health or the result of disputes between central and local authorities? Fuller account of the case studies would help bolster the author’s arguments.”

**Responses**: I have expatiated on the Philippines’ case study so that the narrative is not gravitated towards India. The pilot project in Ilocos Norte was implemented with technical assistance from the US. It did not exclusively on administering BCG vaccinations but relied on a three-pronged strategy, i.e. (a) administering tuberculin tests and vaccinating the detected negative reactors to the tuberculin test, (b) detecting the disease through radiography and (c) treating the infected individuals with isoniazid or streptomycin.  

In the case of Burma, the bureaucratic hassles faced by SEARO officials in getting the tuberculosis  prevalence survey sanctioned was a part of the Burmese government’s attempts to assert its sovereignty in health matters. This was a very helpful suggestion and I have incorporated it while narrating the Burmese experience of TB control during the 1950s.

Additionally, in the case of Indonesia, I have also added on how TB shaped nationalist imagination and the consequent mobilisation mentality. For Burma, I have expatiated on how U Nu’s Pyidawtha ideology shaped the Burmese approach to disease. In the Philippines, the nationalist rhetoric was less explicit although there was an added emphasis on the economic aspects of TB. I have also added a fresh account with respect to the inner tensions between magic bullet approach and a more holistic approach that linked health with nation building. Examples provided not only from India (original version) but also from Indonesia, and the Philippines. In the case of Burma, most of the inner tensions in public health could be teased out from a fuller use of *SEARO Regional Director’s Annual Reports* rather than the voice of local physicians.

**George Dehner, Comment 3:** Expanding on the political aspects of TB control, and clarifying the unifying elements in their nationalist formation and tightening up the transitions between sections will go a long way in making the article more effective.

**Response:** I have rewritten the conclusion. The underlining unifying feature of TB control in India and Southeast Asia (the three examples provided) is the notion of fractured sovereignties. I have spelt out the administrative fractures in great detail in the conclusion and to shore up the argument, have provided briefly how administrative fractures have impeded TB control to this day, with reference to WHO End TB strategy. I have also smoothed the transitions between sections so that the narrative is cohesive.

# Peer review: Referee response for version 1

**DOI:** 10.21956/wellcomeopenres.11362.r20064

**Published:** 2017-02-09

**Authors:** Susan Heydon

**Affiliations:** 1 School of Pharmacy, University of Otago, Dunedin, New Zealand

Tuberculosis remains a global health problem with many challenges facing its control, both related to the disease and the wider context. While increasing scholarship now exists about tuberculosis control in many parts of the world in countries post-independence, this paper aims to add a new approach offering a transnational history of tuberculosis control across South and Southeast Asia. Other themes relate to stigma and private philanthropy. The author discusses anti-tuberculosis campaigns in India, Indonesia, the Philippines, and Burma between 1948 and 1960, particularly exploring their links with discourse about nation building. The very different case studies in this paper pinpoint the limits of national and international public health interventions; they also highlight the importance of the local context in an age post Second World War of enthusiasm for the possibilities of international public health policies and programmes and reliance on technology. In so doing we are reminded of the need to look beyond a narrow biomedical approach to public health problems. Tuberculosis is the issue in this paper, but the point is relevant more broadly.

 Considerable research has been undertaken using a wide range of archival sources and a transnational approach has the potential to offer valuable insights. My reservation is not with the research and the overall argument, but with the way it has been presented. Currently, I find the paper disjointed as it reads as a series of separate examples beginning with India and then moving to Southeast Asia. The balance of the content is also weighted towards India and this I find lessens the impact of the other three and also interesting case studies. Should the title include ‘Southeast Asia’ rather than the more enigmatic ‘beyond’?

 Transnational as a term has different understandings and I note that the abstract does not mention ‘transnational’, although the paper does. I think, however, that such an approach looking across borders could be useful in pulling the paper together. International public health operated within a world of nation states. The Philippines is a member of the Western Pacific region the other three are in the South-East Asia region. Could something be said more broadly about the fluid regional WHO environment at this time and nation building discourse?

 Lastly, a comment on a point made in the conclusion – rather than governments being ‘forced’, the use of ‘magic bullets’ (technology) was often easier and for many preferred; it also avoided having to deal with harder underlying social and economic issues.

 Some specific points:

1. TB is the leading cause of death worldwide from *infectious disease* – not overall.
2. Some of the sentences are much too long – for example on page 3 ending with footnote 15.

I have read this submission. I believe that I have an appropriate level of expertise to confirm that it is of an acceptable scientific standard, however I have significant reservations, as outlined above.

# Response

**Published:** 2018-04-11

**Authors:** Vivek Neelakantan

**Affiliations:** IIT MADRAS, India

**Susan Heydon, Comment 1:** “The paper is disjointed and reads as a series of separate examples beginning with India and then moving to Southeast Asia. The balance of the paper is weighted towards India and this I find, lessens the impact of the other three and also interesting case studies. Should the title include “Southeast Asia” rather than the more enigmatic beyond?”

**Response:** I have substituted the more ambiguous “beyond” with Southeast Asia in the title to better reflect on the scope of the paper. I have also expatiated TB control in Indonesia, the Philippines and Burma so that the narrative is not biased towards India. I have also tightened transitions between paragraphs to improve the flow of the narrative.

                                                                                                                 

**Susan Heydon, Comment 2:** “Transnational, as a term has different understandings. I note that the abstract does not mention ‘transnational’ but the paper does. I think that such an approach looking across borders could pull the paper together. The Philippines is a member in the West Pacific region, the other three in the Southeast Asia region. Could something be said about fluid regional WHO environment at this time and nation-building discourse?

**Response:** I have emphasised “transnational” in the abstract of the paper to spell-out the scope of the paper. I have emphasised the birth of the SEARO and WPRO (WHO Regional Offices of Southeast Asia and West Pacific) to emphasise how the WHO, in the 1950s, supported disease eradication programs through pilot health demonstration projects (in this case TB). The regionalisation of the WHO has provided better context to the narration of TB Control projects in Indonesia, the Philippines, and Burma. I have spelt out the framing of TB in nationalist imagination in Indonesia (L.G.J. Samallo’s interpretation) and Burma ( *Pyidawth*a). Although data for the Philippines is not available, I have spelt out the rhetoric around TB in the Philippines at the time, framed in terms of poverty.

**Susan Heydon, Comment 3:** Rather than being forced, the use of magic bullets was often easier.

**Response: **I have co-opted the reviewer’s comment in the conclusion.

**Susan Heydon, Comment 4 (a)** TB is the leading cause of death worldwide, not overall.

**Response:** I have co-opted the suggestion. The statement has been revised in accordance with the latest WHO statistics on TB (2017).

Susan Heydon, Comment 4 (b) V.1. Some sentences are too long, for e.g. page 3, footnote 15

Response: I have split the sentences for clarity. For purposes of smooth flow of narrative, I have split the long-winding sentences into simpler sentences for enhancing the clarity of the paper. Page 3, footnote 15 of V. 1.  The introduction of Rajaji and A.V. Raman moved to the footnote for improving flow of the main text. Page 6, of V2.

# Peer review: Referee response for version 1

**DOI:** 10.21956/wellcomeopenres.11362.r19924

**Published:** 2017-02-06

**Authors:** Liping Bu, Shirish N. Kavadi

**Affiliations:** 1 Department of History, Alma College, Alma, MI, USA; 2 Symbiosis International University, Pune, Maharashtra, India

The study of tuberculosis control is usually conducted about the efforts of individual countries to evaluate the outcome and draw lessons. In this article, Vivek Neelakantan tries to break the boundaries of individual nations to draw out certain patterns of TB control campaigns in four different countries of South and Southeast Asia, namely India, Indonesia, the Philippines, and Burma. The focus of analysis is on the politics of anti-BCG vaccination in South India, Madras state to be more precise, where local politicians spread and exploited popular fear of the side-effect of the vaccine for their own political agenda to counter the central control of the government in New Delhi. Private physicians were another major group of power who opposed the vaccination campaign, as they were concerned that the BCG vaccination would reduce their lucrative medical practice. The major proponents for the BCG vaccination were the national governments and international organizations such as WHO, UNICEF and ITC. These actors regarded the TB control, and disease prevention in general, as an integral part of nation-building and improvement of human lives. In his study, Neelakantan finds that there were various tensions existing between the local implementation and the central planning. There were also tensions between the narrow biomedical approach that targeted one individual disease such as TB that was promoted by international organizations and the holistic approach that targeted diseases with broader commitment to socioeconomic improvement in nation-building by post-colonial governments.

 The author states that his study situates TB control within the broader context of international health, but the article does not provide a clear picture of international health in either the South Asia/Southeast Asia region or even broader context. A specific discussion of the context of international health will also help to clarify how implementation of TB control in South Asia/Southeast Asia sheds light on the nature of post-colonial state sovereignty in public health. The author asserts that by discussing TB campaigns in the four countries the article links their political history of decolonization to the history of international health. This part of the article is weak since the political history of decolonization in these countries has not been discussed. In this regard, an examination of the nationalist discourse in the four countries and the local states of India on preventive medicine and TB control will help readers understand the politics of public health and nation-building in that region. In doing so, the author may want to re-define the title of the article to better reflect the content.

 In the discussion of regional differences within India (p. 5), it would help if the author explained, if data available, the differences in incidence and prevalence rates in West Bengal and Travancore and Cochin (present day Kerala) and how people viewed TB before writing about the different responses to the BCG vaccination campaign. Incidentally, Bengal and Travancore also have rice based diets. Did similar perceptions exist in these two provinces with respect to prevalence rates and diet? In terms of people’s view and understanding of disease and prevention, it is helpful to provide a brief historical account of public health in these different regions. Kerala today is viewed as a model with respect to public health in India mainly due to its unique modern history. Travancore was a Princely State and not part of British India. The Rockefeller Foundation was deeply involved in developing public health programs and the public health department in that state. 

 As to the change of Rajaji’s stand after he took over as Chief Minister of Madras state, it is not clear whether the change was a result of Raman’s campaign of popular sentiment or of Rajaji’s opposition to Delhi’s centralizing tendencies or a combination of both. A further clarification or elaboration would help. Were the nationalist anxieties that Raman and Rajaji had about Indians being used as experimental subjects shared by others in India either in official and non-official circles and by professionals and non-professionals? A broader Indian context will help explain the question. Kavadi’s “Medicine, Philanthropy, and Nationhood” in Public Health and National Reconstruction in Post-War Asia (Bu and Yip, 2015) would enrich the argument.

 The “fractured sovereignties”, as Neelakantan terms them, were, in fact, of many different natures. Some were political fights between the local and the central governments, some were inadequacies of administrative capability and deficiency of supplies, and others were tensions in the negotiation of sovereignty over disease control between international demands and national necessities. For instance, in Madras it appeared more political, with Raman and Rajaji each promoting their own agenda; whereas in Burma and the Philippines the fractures were located within the bureaucracy or associated with technical difficulties. The author needs a bit more analysis of the fractures in health policy implementation and post-colonial health sovereignties. The idea of ‘fractures’ has been discussed by other scholars with reference to various levels, entities, actors in the political and administrative structure and system, namely, Bhattacharya, Harrison and Worboys (2005), Sanjoy Bhattacharya (2006), Ryan Johnson and Amna Khalid (2011), and Kavadi (2015 & 2016).

 The author could have gone into a bit more details to discuss Indonesia, the Philippines and Burma. It would be useful to know how different national governments handled the dissemination of knowledge about disease and the TB control. In other words, did the governments do a good job in popular education about TB and its prevention and treatment? One also wishes that the author elaborated more on whether the South India opposition to BCG vaccine stood out as a unique case or a representative example of anti-TB campaigns in India in general. It may be useful to look at Margaret Jones’ article (2016) on TB control in Sri Lanka during 1948-1990, which was not about BCG but a community-oriented control model that also faced obstacles and resistance in implementation.

We have read this submission. We believe that we have an appropriate level of expertise to confirm that it is of an acceptable scientific standard, however we have significant reservations, as outlined above.

# Response

**Published:** 2018-04-11

**Authors:** Vivek Neelakantan

**Affiliations:** IIT MADRAS, India

**Liping Bu and Shirish Kavadi, Comment 1:**  The article does not provide a clear picture of international health in either South or Southeast Asia. A specific discussion on international health will also clarify how TB control in Southeast Asia sheds light on the nature of postcolonial sovereignty.

**Response:** I have contextualised the pilot TB control projects in each of the countries within the framework for the emergence of the WHO and establishment of its regional offices in New Delhi and Manila. I have also eliminated the tenuous link between decolonisation and TB control, given the lack of data and have instead reshaped the argument in favour of fractured sovereignties, a recurring theme in all four countries and how these administrative fractures have undermined TB control to the present day (a point illustrated in the conclusion).

Liping Bu and Shirish Kavadi, Comment 2: Change of Rajaji’s strategy towards BCG after he took over as Chief Minister?

**Response: **The context was political. He opposed the centralising tendencies of Delhi. Domestically, his educational reforms were controversial as his opponents denounced him for promoting *kula kalvi thittam*, or caste-based hereditary occupation through his educational strategy. As Niels Brimnes pointed out, Rajaji did not have an alternative to state-medicine (BCG) unlike his friend A.V. Raman, the editor of *People’s Health*.

**Liping Bu and Shirish Kavadi, Comment 3:** “Fractured Sovereignties as Vivek Neelakantan points out, were in fact of many different natures.”

**Responses: **A helpful observation and I have underlined the fractures as the key theme in the conclusion of V. 2 of the article. Fractured sovereignties in Madras were more political. While discussing Madras, I have highlighted the fight within Madras itself between competing priorities of the state government, in a separate paragraph dedicated to T.S.S. Rajan, Minister of Health of Madras, and a colleague of the Minister of Health A.B. Shetty.

I have expatiated the argument on the notion of fractured sovereignties in the conclusion. The recommending readings Sanjoy Bhattacharya’s *Expunging Variola*, and Shirish Kavadi’s chapter published in Liping Bu and Ka-Che Yip’s edited volume were very helpful for strengthening the argument. I did not consult *Fractured States*, as I have deleted the tenuous assertion earlier made between decolonisation and TB control. Margaret Jones’ article is helpful to understand the shortcomings of WHO-sponsored anti-TB campaign in Sri Lanka grafted onto the extant health system. I have incorporated Margaret Jones’ analysis in the paragraphs focusing on extant historiography. In the conclusion, I have clarified that the South Indian opposition to BCG, spearheaded by A.V. Raman was hardly unique in the history of BCG campaigns across Asia. In the Philippines, similar issues were raised with respect to the vaccine’s efficacy. But, the opposition to BCG was steamrolled only to resurface by the mid-1950s when Filipino paediatricians reported complications, a point also shared by India and Indonesia.

**Liping Bu and Shirish Kavadi, Comment 4**:  “ In discussion of regional differences within India, it would help if the author explained, if data available , the differences in incidence and prevalence rates in West Bengal and Cochin (present-day Kerala) and how people viewed TB before writing about the different responses to the BCG vaccination campaign?”

**Responses:** Unfortunately, data for incidence and prevalence rates for TB in Bengal and Cochin, for the early 1950s is not available. I have also seriously taken into account the observation of Shirish Kavadi that the Rockefeller Foundation’s work in Travancore was instrumental in shaping the favourable public opinion of the local press in Trvancore and Cochin states after Indian independence, towards BCG. While this is true, the Rockefeller Foundation did not concentrate on TB control in India during the 1950s (the focus was on the Caribbean, instead). For e.g. Henrice Altink’s article in *Medical History*. Therefore I have not mentioned the Rockefeller Foundation’s role in shaping public opinion in Cochin and Travancore towards BCG, for the early-1950s. Given the vastness of India, comparable to a “continent,” and not a country, in the view of a WHO technical report, I found it feasible to concentrate on one part of India, i.e. Madras state for which I had locally-available archival materials.

# Peer review: Referee response for version 1

**DOI:** 10.21956/wellcomeopenres.11362.r19311

**Published:** 2017-02-03

**Authors:** Christian W. McMillen

**Affiliations:** 1 Cocoran Department of History, University of Virginia, Charlottesville, VA, USA

Let me note from the outset that the author’s research project and the questions he is asking combined with the sources he is interrogating—such as the Indonesian language primary documents I doubt many others have ever examined—have the potential to broaden historians’ understanding of the post-World War II global health landscape. For the author is doing research on areas largely ignored by others; he is asking vital questions of critical importance to many scholars working in the field. 

 The present article—well conceived, though not always well executed—attempts to link the history of anti-tuberculosis campaigns in India, Burma, Indonesia, and the Philippines. The author has set for himself an ambitious agenda that is partially fulfilled. While I admire the effort to connect the history of TB control in these four countries as a way to make an argument about decolonization in the post-World War II era the author will need to do more work to make his claims less subject to criticism. To my mind there are too many unsubstantiated arguments. The author’s principle argument is that “Resistance to vaccination in postcolonial contexts stems from the association of the vaccine with the colonial state’s interventionist ambitions and nationalist critique of health policies of the colonial  government.” However, the argument is well substantiated only for India where a significant anti-BCG vaccination campaign stymied progress in the south. In Indonesia, on the other hand, while the author makes clear that the BCG campaign faced barriers from the start—“organisational shortcomings of the TB control programme”; “passive resistance from the Chinese community”; and complaints that BCG caused fevers—it is not clear that any of this had anything to do with resistance to the colonial or postcolonial state. The connections in the Philippines and Burma are even more tenuous. In Burma, the author notes, there was a high defaulter rate at the Rangoon TB clinic. Why is this not explored. The reader is left to assume that the details regarding the high defaulter rate are offered in support of the author’s overall claims about resistance to the colonial and post-colonial state. But I don’t see the connection.

 As noted above, in the conclusion the author writes: “Resistance to vaccination in postcolonial contexts stems from the association of the vaccine with the colonial state’s interventionist ambitions and nationalist critique of health policies of the colonial government.” This is a provocative and compelling hypothesis and one that the author soundly argues was the case in South India. But the examples from Indonesia, Burma, and the Philippines do not support it. This does not make the claim incorrect; it does point to the need for the author to shore up the claims. This could be done in a longer piece or one of similar length that does not give such lopsided coverage to India—the section on India is longer on its own than the section covering the other three countries. Further on in the conclusion the author writes, “This study reveals the benefits of linking the history of tuberculosis in Southern India and Southeast Asia to the broader question of decolonisation in the aftermath of World War II.” I would agree that benefit could come from linking the history of TB to the history of decolonization. I am thus eager to see how the author will make that connection less tenuous in future publications.

I have read this submission. I believe that I have an appropriate level of expertise to confirm that it is of an acceptable scientific standard, however I have significant reservations, as outlined above.

# Response

**Published:** 2018-04-11

**Authors:** Vivek Neelakantan

**Affiliations:** IIT MADRAS, India

**Christian McMillen, Comment 1:** To my mind, there are too many unsubstantiated arguments. The author’s principle argument is that resistance to vaccination stems from the association of the vaccine with the colonial state’s interventionist ambitions and nationalist critique of health policies of the colonial government.”

**Response:** I have deleted the unsubstantiated claim as historiography/ primary sources were deficient for Burma (Myanmar). The scope of the paper is limited to TB control in the first decades of independence. The contention about decolonisation and TB control was also tenuous due to the primary sources skewed in favour of India. Instead of focusing on the tenuous linkages between decolonisation and TB control, I have revised the argument. I have expatiated on the notion of fractured sovereignties, an underlining theme impeding TB control in India and Southeast Asia. Fractured sovereignties existed at different levels, a point I have highlighted in the conclusion. I have also highlighted how administrative fractures in public health have affected the WHO’s End TB Strategy towards the end of the paper.

**Christian McMillen, Comment 2:**  The author notes that the BCG campaign faced organisational barriers from the start. For e.g. passive resistance of the Chinese community, complaints that the BCG vaccine had caused fevers. But, this had nothing to do with the resistance to the postcolonial state. Examples from Indonesia, Burma, and Philippines, do not support it.

**Response:** I agree with your comment. Given the bias of secondary literature towards India, I had to rewrite the argument in favour of administrative fractures. I have expatiated on the BCG campaigns in Philippines and reservations of local physicians, the way the government steamrolled on opposition toward BCG (early 1950s), and the recrudescence of opposition, led by Filipino paediatricians towards the mid-1950s when it was alleged that vaccination led to the death of infants. I have rewritten entire paragraphs on Burma so that the scope of the paper is tightened to reflect on the time-frame between 1948 and 1960, when U Nu’s Pyidawtha ideology reigned influential and shaped the political culture of health. I have deleted the references to high rate of default at the TB clinic in Rangoon (1964) as it falls beyond the scope of the article. I have instead, focused on administrative fractures that had a decisive influence on the momentum of the anti-TB campaign in Burma.

**Christian McMillen, Comment 3:** “This study reveals the benefits of linking the history of TB control in South India and Southeast Asia to the history of decolonisation. I am thus eager to see how the author will make that connection less tenuous in future publications?”

I have deleted the tenuous claim due to lack of adequate archival sources for countries in Southeast Asia. Instead, I have highlighted the tensions between the notional magic bullet and a more comprehensive approach that linked health to broader questions (as highlighted in the BCG controversy in India, the Philippines, and the nationalist framing of TB in Indonesia, added in the revised version). Also, the notion of fractured sovereignties offers an invaluable insight to knit together the history of TB in disparate international contexts.
